# Chemical‐Shift Perturbations Reflect Bile Acid Binding to Norovirus Coat Protein: Recognition Comes in Different Flavors

**DOI:** 10.1002/cbic.201900572

**Published:** 2019-12-05

**Authors:** Robert Creutznacher, Eric Schulze, Georg Wallmann, Thomas Peters, Matthias Stein, Alvaro Mallagaray

**Affiliations:** ^1^ University of Lübeck, Center of Structural and Cell Biology in Medicine (CSCM) Institute of Chemistry and Metabolomics Ratzeburger Allee 160 23562 Lübeck Germany; ^2^ Max Planck Institute for Dynamics of Complex Technical Systems Molecular Simulations and Design Group Sandtorstrasse 1 39106 Magdeburg Germany

**Keywords:** chemical shift perturbation, ensemble docking, long-timescale MD, molecular recognition, STD NMR spectroscopy

## Abstract

Bile acids have been reported as important cofactors promoting human and murine norovirus (NoV) infections in cell culture. The underlying mechanisms are not resolved. Through the use of chemical shift perturbation (CSP) NMR experiments, we identified a low‐affinity bile acid binding site of a human GII.4 NoV strain. Long‐timescale MD simulations reveal the formation of a ligand‐accessible binding pocket of flexible shape, allowing the formation of stable viral coat protein–bile acid complexes in agreement with experimental CSP data. CSP NMR experiments also show that this mode of bile acid binding has a minor influence on the binding of histo‐blood group antigens and vice versa. STD NMR experiments probing the binding of bile acids to virus‐like particles of seven different strains suggest that low‐affinity bile acid binding is a common feature of human NoV and should therefore be important for understanding the role of bile acids as cofactors in NoV infection.

## Graphical abstract

**Soft recognition**: Bile acids are known to promote norovirus infection, but the molecular basis is unknown. NMR spectroscopy and molecular dynamics simulations join forces to characterize bile acid binding to norovirus coat proteins, suggesting promiscuous low‐affinity binding contributes as a potential trigger mechanism.

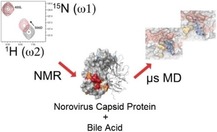

## Introduction

Norovirus infections are the leading cause of viral gastroenteritis infections worldwide.^1^ There is compelling evidence that attachment of human noroviruses to histo‐blood group antigens (HBGAs) is essential for infection,^2^ and therefore, the HBGA binding site located in the protruding domain (P‐domain) of the human norovirus capsid protein VP1 has been targeted to identify potential entry inhibitors.^3^ One of the major bottlenecks in norovirus research has been the lack of a human norovirus cell culture system. The development of a mouse norovirus infection model and of corresponding cell culture systems has been a significant success but was not transferable to human noroviruses. During the past years human norovirus cell culture systems have become available paving the way for many functional studies. It turned out that besides HBGAs bile acids represent another important cofactor for the promotion of norovirus infection.^4^

Two crystallographic studies addressed the binding of bile acids to human and to murine norovirus capsid protein.4a, 4c For particular strains of human NoVs two symmetrical bile acid binding pockets with affinities in the low‐micromolar range have been identified adjacent to the HBGA binding sites.4a In the case of a rare GII.1 strain, binding of bile acids promoted attachment to HBGAs. Surprisingly, for the dominant epidemic causing GII.4 and GII.17 strains no ligand binding to these pockets was observed, leaving open the question of why bile acids are essential for replication of GII.17 viruses and significantly promote replication of GII.4 viruses in human intestinal enteroids.4f Another study based on X‐ray crystallography and cryo‐electron microscopy has identified two symmetrical bile acid binding pockets at the P‐domain dimer interface of a CW3 mouse NoV.4c Interestingly, these binding pockets are highly specific for glycochenodeoxycholic acid (GCDCA) with low‐micromolar affinity. For human NoVs inspection of available crystal structures of P‐domains shows that this binding pocket is inaccessible to bile acid molecules.

Long‐timescale molecular dynamics (MD) simulations can reveal transient ligand binding pockets tailored for small molecule binding.^5^ A ligand may selectively bind to one or to an ensemble of such pre‐existing conformations.^6^ The sites of binding are referred to as sub‐pockets, adjacent pockets, breathing motion, channel/tunnel, or allosteric pockets.^7^ MD simulations sample conformational space and provide snapshots of relevant protein conformations for docking, improving the accuracy of virtual screening over rigid protein docking.^8^ The method also facilitates discovery of allosteric sites.^9^

**Scheme 1:**
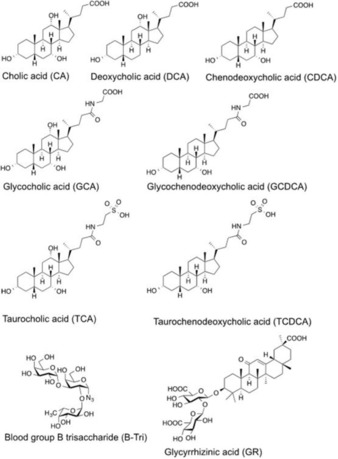
Chemical structures and abbreviations of ligands used for NMR experiments.

Recently, we obtained a nearly complete backbone assignment of the P‐domain of a human epidemic genotype norovirus, GII.4 Saga.^10^ We also obtained a complete methyl group assignment of a MILVA‐labeled sample of GII.4 Saga P‐domain (unpublished data). Based on this work, it is now possible to investigate ligand binding using protein NMR experiments. It is well established that chemical shift perturbation (CSP) NMR experiments either based on ^1^H,^15^N TROSY HSQC or on methyl TROSY experiments provide exhaustive information on ligand binding sites under near‐physiological conditions.^11^ Therefore, we used CSP NMR experiments to study bile acid binding to human GII.4 Saga P‐domains uncovering a low‐affinity binding site for bile acids (cf. Scheme 1). This site is not present in published crystal structures but becomes ligand‐accessible during long‐timescale MD trajectories. Ensemble‐based docking of various bile acid molecules to a large number of conformations plus additional MD refinement of high‐ranked poses reveals the plasticity of this site, yielding binding poses in very good agreement with CSP data. Finally, STD NMR experiments using VLPs of seven different human NoV strains suggest that lowaffinity bile acid binding is a common feature of human NoVs.

## Results

### CSP NMR experiments identify a bile acid binding pocket

**Figure 1:**
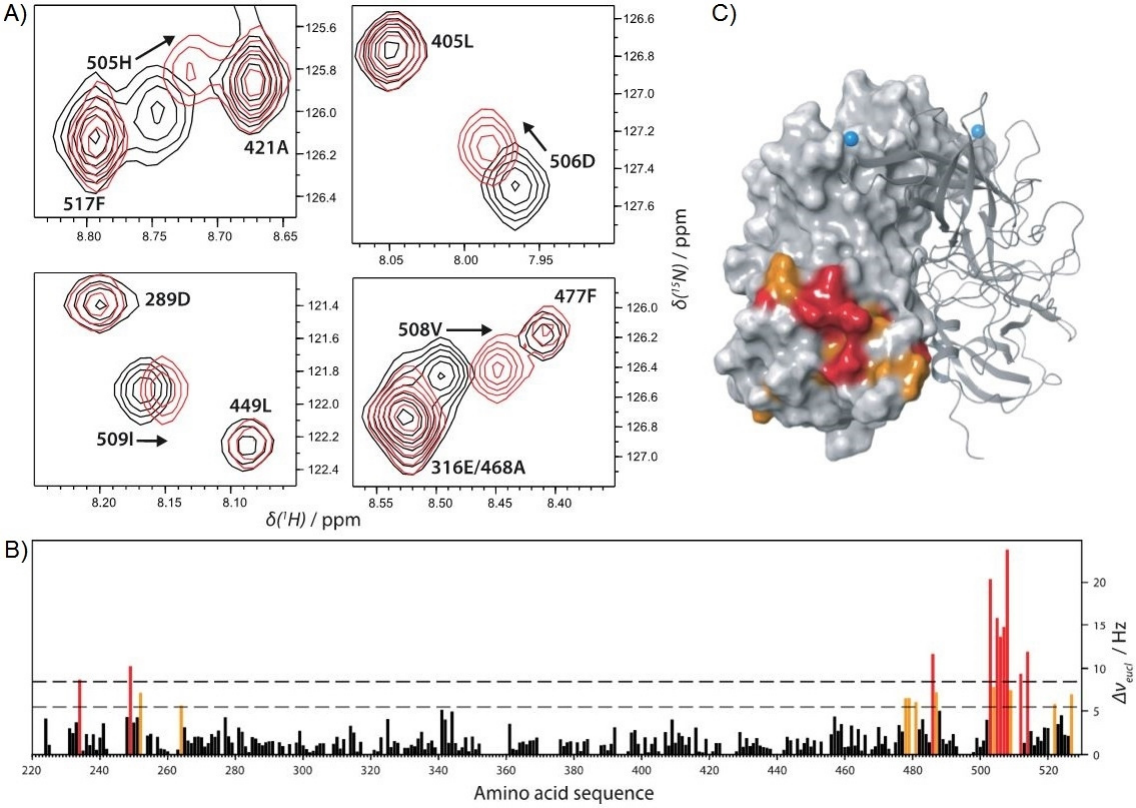
A) Regions of a ^1^H,^15^N TROSY HSQC spectrum showing backbone NH signals of a [U‐^2^H,^15^N] labeled sample of P dimers of GII.4 Saga norovirus (100 μm) being disturbed by the presence of 8 mm cholic acid (CA). The spectrum was recorded at 500 MHz and 298 K. B) Chemical shift perturbations (CSPs, calculated as Euclidean distances) of backbone NH signals as a function of amino acid position. CSPs larger than mean+*σ* are shown in orange, and values larger than mean+2 *σ* in red. C) Mapping of CSPs onto the crystal structure of P dimers (PDB ID: https://www.rcsb.org/structure/4X06) using the color coding in panel (B). The remote HBGA binding site is highlighted with a blue ball (position of C6 of the fucose moiety of B‐trisaccharide).

Binding of bile acids to the P‐domain of VP1 of GII.4 Saga NoV was studied by using ^1^H,^15^N TROSY HSQC spectra as well as methyl TROSY spectra, identifying perturbations of backbone NH signals and of side chain methyl groups, respectively. Samples were uniformly ^2^H,^15^N‐labeled ([U‐^2^H,^15^N]) or specifically ^13^C‐methyl (MIL^ProS^V^ProS^A)‐labeled. Binding of four bile acids, CA, DCA, GCDCA, and CDCA (Scheme 1) was tested with backbone ^1^H,^15^N HSQC TROSY experiments (Figure 1 and Figure S1 in the Supporting Information). For comparison, CA‐induced CSPs were also derived from a methyl TROSY experiment (see below). Both types of CSP experiments yielded two symmetrical binding sites close to the C termini of the dimeric P‐domain (P dimer) and at large distance to the HBGA binding pockets as this is shown in Figure 1 C for CA as representative example.

Binding of DCA, GCDCA, and CDCA yields very similar CSPs reflecting binding to the same site (cf. Figure S1). NMR signals of amino acids in the HBGA binding pocket or at the sites matching the high‐affinity bile acid binding pockets reported for rare genotypes of human NoV4a or murine NoV4c remain unaffected.

### STD NMR experiments demonstrate bile acid binding to P dimers and to VLPs from different norovirus strains

Using STD NMR experiments, we tested binding of CA, GCA, GCDCA, TCA, and TCDCA to viral capsids. For these experiments we used VLP samples from different laboratories and from different NoV strains (GI.1 Norwalk, GII.4 Saga, GII.4 Ast6139, GII.7 RKI, GII.10 Vietnam026, GII.17 Kawasaki308, and GII.17 Saitama/T87). In each case STD NMR spectra indicate binding of bile acids to VLPs (Figure S2). The STD NMR spectra did not indicate any significant differences across the strains suggesting similar binding modes. Therefore, we chose CA as a representative bile acid for further STD NMR experiments.

**Figure 2:**
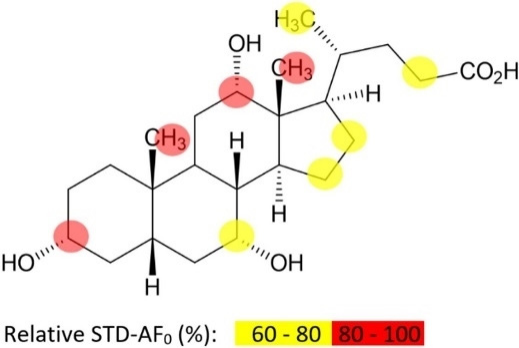
Binding epitope of CA bound to GII.4 VLPs from STD NMR buildup curves (cf. Figure S3). Almost all protons receive saturation, but due to overlap STD amplification factors (AF) could only be determined for a subset of protons. Where STD amplification factors could be obtained respective protons are color coded. Experiments were performed at 600 MHz, with the temperature set at 277 K.

For a sample of GII.4 Saga VLPs we recorded STD buildup curves and determined a binding epitope (Figure 2) from initial STD growth rates, complementing the topological information about the binding site from CSP NMR (Figure 1). Almost all CA protons receive saturation from the protein but due to signal overlap STD amplification factors were obtained only for a subset of protons. The corresponding binding epitope suggests that one side of the steroid skeleton makes closer contact with protons located in the binding pocket.

### NMR titration experiments provide relative affinities for CA binding

We performed CSP and STD NMR titration experiments to study the binding of CA and GCDCA to NoV P dimers and VLPs. CA was chosen, as its solubility in water is higher than other bile acids. GCDCA is less water soluble, but was included because GCDCA has been the focus of previous investigations.4a, 4c

**Figure 3:**
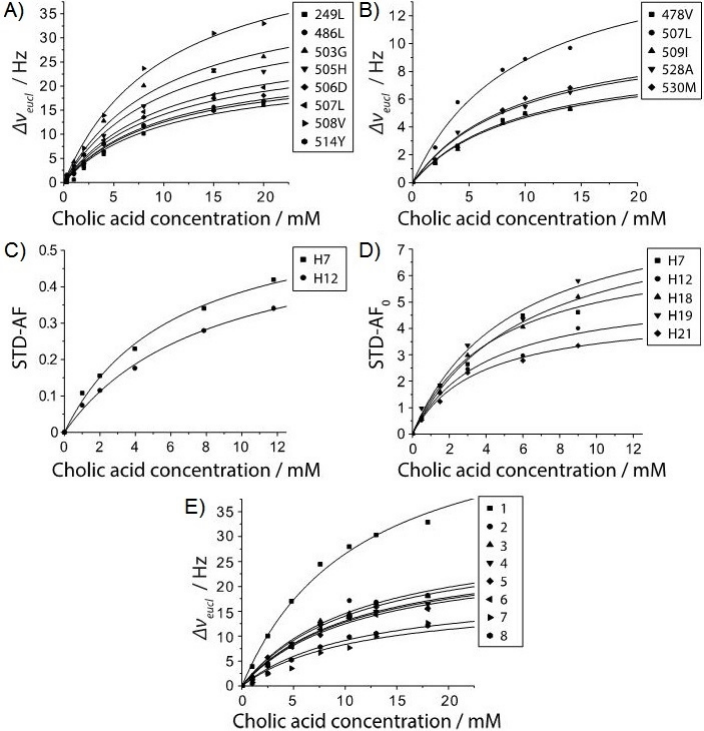
Binding isotherms from NMR titration experiments using cholic acid as ligand. A) Binding isotherms from CSPs in ^1^H,^15^N TROSY HSQC spectra of GII.4 Saga P dimers. B) Binding isotherms from CSPs in methyl TROSY spectra of GII.4 Saga P dimers. C) Binding isotherms from STDs in the presence of GII.4 Saga P dimers. D) Binding isotherms from initial growth rate STDs in the presence of GII.4 Saga VLPs (cf. Figure 2). E) Binding isotherms from CSPs in ^1^H,^15^N TROSY HSQC spectra of GII.4 MI001 P dimers.

It is well established that bile acids form micelles when dissolved in water above a critical concentration.^12^ These micelles differ from conventional micelles in that the micellar assembly consists of only few molecules (<10–20).^13^ Formation of such aggregates has been reported to begin at higher concentrations for CA than for DCA or TCA, as this is reflected by critical micelle concentrations of about 5 mm obtained from diffusion ordered NMR experiments.^14^ From chemical shift changes observed in simple ^1^H NMR spectra of CA and GCDCA at increasing concentrations we conclude that the formation of aggregates begins at concentrations above about 4 mm for CA, and above about 1 mm for GCDCA (cf. Figure S4). Therefore, interpretation of binding isotherms (Figure 3) requires caution. Our data may well reflect binding of free CA or GCDCA molecules and of aggregates at the same time. On the other hand, exchange between free and micelle‐associated bile acid molecules should be rapid, not limiting the amount of free bile acid molecules binding to P dimers. Nevertheless, analysis of binding isotherms cannot provide true dissociation constants as saturation is only reached at ligand concentrations well above estimated critical micelle concentrations, making an assessment of the contribution of aggregates impossible. Consequently, we only report *apparent* dissociation constants and translate these values into relative affinities for meaningful comparisons (Table 1). Of note, all apparent dissociation constants were of the same order of magnitude in the low‐millimolar range. Assuming the contribution of aggregation is constant for all titrations with CA, a comparison of relative binding affinities for different strains is possible, with lower relative values indicating increased affinities. The ^1^H,^15^N TROSY HSQC based titration of GII.4 Saga P dimers with CA was used as a reference with the relative affinity set to 1.0 (Table 1, No. 2).

**Table 1:** Relative affinities for binding of CA to NoV P‐domains and VLPs.^[a]^

No.	Ligand	NoV strain	Protein	Isotopic labeling	NMR experiment	*T* [K]	*K*_D app_ [mm]	R^2^	Relative affinity^[c]^
1	CA	GI.1 Norwalk	VLPs	–	STD	277	12.2±1.8	0.9933	1.2
2	CA	GII.4 Saga	P dimers	[U‐^2^H,^15^N]	HSQC TROSY	298	10.0±0.6	0.9924	1.0
3	CA	GII.4 Saga	P dimers	MIL^ProS^V^ProS^A	methyl TROSY	298	9.5±1.1	0.9881	1.0
4	CA	GII.4 Saga N373D	P dimers	–	STD	277	6.2±1.1	0.9929	0.6
5	CA	GII.4 Saga	VLPs	–	STD	277	4.7±0.4	0.9912	0.5
6	CA	GII.4 Saga	VLPs	–	STD	277	3.6±0.7^[b]^	0.9907	0.4
7	CA	GII.4 MI001	P dimers	[U‐^2^H,^15^N]	HSQC TROSY	298	11.1±0.9	0.9882	1.1
8	CA	GII.4 Ast6139	VLPs	–	STD	277	26.5±8.0	0.9830	2.7
9	CA	GII.7 RKI	VLPs	–	STD	277	10.9±4.3	0.9775	1.1
10	CA	GII.10 Vietnam026	VLPs	–	STD	277	12.9±2.9	0.9881	1.3
11	CA	GII.17 Kawasaki308	P dimers	MIL^ProS^V^ProS^A	Methyl TROSY	298	20.1±2.9	0.9895	2.0
12	CA	GII.17 Kawasaki308	VLPs	–	STD	277	31.5±2.8	0.9990	3.1
13	CA	GII.17 Saitama/T87	VLPs	–	STD	277	6.4±0.5	0.9988	0.6
14	GCDCA	GII.4 Saga	P dimers	[U‐^2^H,^15^N]	HSQC TROSY	298	1.5±0.3	–	–
15	GR	GII.4 Saga	P dimers	[U‐^2^H,^15^N]	HSQC TROSY	298	13.8±0.4	0.9977	–

[a] Relative affinities for GCDCA and GR cannot be compared because the contribution of ligand aggregates to binding is different (cf. main text). Samples used for STD experiments and for ^1^H,^15^N TROSY HSQC experiments contained 10 % D_2_O, and the solution was adjusted to pH 7.3. Samples for methyl TROSY experiments contained >99 % D_2_O, and the solution was adjusted to pH 7.4 to create comparable conditions. [b] Apparent dissociation constants were calculated from STD initial growth rates (STD‐AF_0_). [c] Arbitrary units.

For binding of CA to GII.4 Saga P dimers we performed three different and independent types of NMR titration experiments. One set of titrations used CSPs from ^1^H,^15^N TROSY HSQC spectra, a second set of titrations was based on methyl TROSY spectra, and a third data set was based on STD NMR spectra. In all three cases we obtained similar relative affinities (Table 1, No. 2–4; Figure 3). For the STD NMR titration we used the N373D mutant, which cannot undergo deamidation. The experiment was performed at a lower temperature (277 K) than the CSP titrations (298 K), which may account for the slightly lower relative affinity (Table 1, No. 4).

To compare P dimers to viral capsids, we also obtained STD NMR titration curves for CA binding to GII.4 Saga VLPs (Figure 3 D; Table 1, No. 5 and 6). In one set of experiments we performed titrations at single saturation times of 2 s^15^ (cf. Figure S5 and Table S1). Another titration experiment made use of initial STD growth rates^16^ determined from STD build up curves (Figure S6 and Table S2). The initial growth rates were also used to derive a binding epitope for CA (Figures 2 and S3). The apparent *K*_D_ values obtained are similar, with the value resulting from initial growth rates being slightly smaller, as expected.

We also studied binding of CA to [U‐^2^H,^15^N]‐labeled P dimers of a related GII.4 strain (MI001) that infects humans as well as mice.^17^ As we have no backbone assignment for this strain yet, we compared backbone chemical shifts of MI001 P dimers to Saga P dimers, yielding some tentative assignments (Figure S7). Based on these preliminary data, comparison of CSPs measured in ^1^H,^15^N TROSY HSQC spectra suggests that the binding pockets of MI001 and Saga are rather similar, and titration curves (Figure 3) yield a very similar relative affinity compared to Saga (Table 1, No. 2 and 7).

A sample of MIL^ProS^V^ProS^A labeled P dimers of a GII.17 Kawasaki308 strain was also subjected to a methyl TROSY based CSP titration showing specific effects of binding. In this case, assignments are not available yet. Nonetheless, a relative affinity can be obtained from determining CSPs of unassigned cross‐peaks (Table 1, No. 11, Figure S8).

STD NMR titrations were performed for VLPs of the NoV strains GII.4 Ast6139, GII.7 RKI, and GII.10 Vietnam026, GII.17 Kawasaki308 and GII.17 Saitama/T87 as well as for VLPs of the GI.1 Norwalk virus, using a single saturation time of 2 s (Table 1, No. 1, No. 8–10, and No. 12–13). Relative affinities for GI.1 Norwalk, GII.10 Vietnam026, GII.7 RKI, GII.17 Saitama/T87 and GII.4 Saga VLPs are very similar. For GII.4 Ast6139 and for GII.17 Kawasaki308 VLPs as well as for Kawasaki308 P dimers slightly larger relative affinities are found.

To study binding of GCDCA to GII.4 Saga P dimers we finally determined an apparent dissociation constant from a CSP titration based on ^1^H,^15^N TROSY HSQC spectra (Table 1, No. 14). However, the apparent dissociation constant derived has to be treated with great caution, as only the Val508 NH signal could be used for calculating a dissociation constant (Figure S9). All other CSPs were too low to justify global fitting. Moreover, as discussed above, this value cannot be directly compared with the CA titrations, and, therefore, no relative affinity is reported in Table 1.

### Deamidation of Asn373 does not affect binding of CA

**Figure 4:**
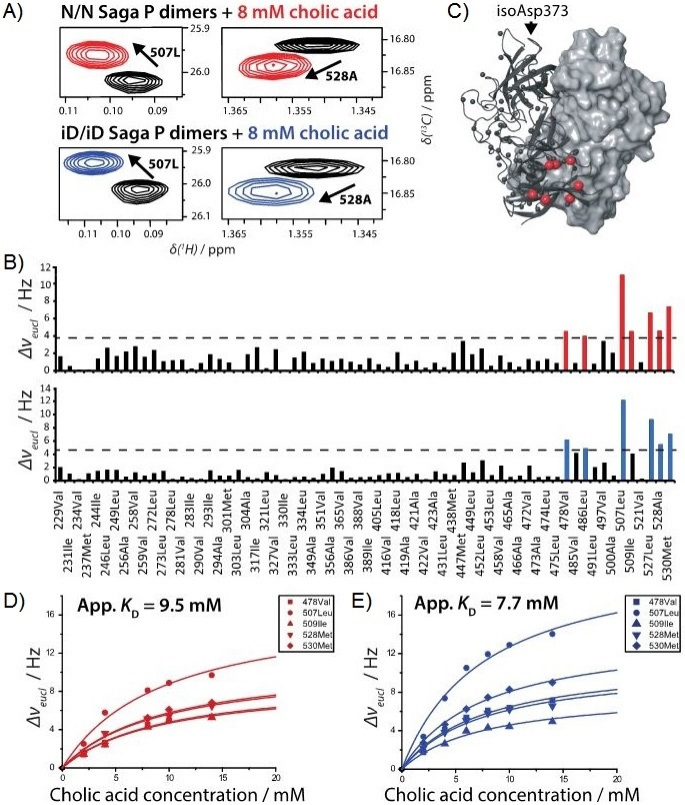
A) Regions of methyl TROSY spectra of a ^13^C‐methyl (MIL^ProS^V^ProS^A)‐labeled sample of GII.4 Saga NN and iDiD P dimers. Two representative cross‐peaks demonstrate that perturbations upon addition of CA are unaffected by deamidation of Asn373. B) CSPs as a function of amino acid position. CSPs at 8 mm CA larger than mean+*σ* (‐ ‐ ‐ ‐) are color coded (red: NN P dimers; blue: iDiD P dimers). C) ^13^C methyl CSPs mapped onto the surface of GII.4 Saga NN P dimers (PDB ID: https://www.rcsb.org/structure/4X06). The deamidation site Asn373 is highlighted. D) Binding isotherms from chemical shift titrations of NN P dimers with CA. E) Binding isotherms from chemical shift titrations of iDiD P dimers with CA.

For GII.4 Saga P dimers it has been shown that spontaneous deamidation of Asn373 and subsequent formation of an isoaspartate residue at this position abrogates HBGA binding.^10^ This process is likely to be relevant for about 66 % of all GII.4 strains. Therefore, we tested the influence of deamidation on CA binding to GII.4 Saga P dimers. Native Saga P dimers (NN P dimers) and completely deamidated Saga P dimers (iDiD P dimers) were purified using an ion‐exchange chromatography protocol and immediately subjected to CSP NMR experiments, keeping conversion of NN P dimers at a minimum. Comparison of CSPs in corresponding methyl TROSY spectra upon titration with CA demonstrates that deamidation has practically no influence on binding. Almost identical apparent *K*_D_ values were obtained (Figure 4).

We also applied STD NMR titrations to compare binding of CA to the N373D mutant of GII.4 Saga P dimers. This mutant does not undergo deamidation and does not convert into the iso‐aspartate form. Within experimental error, the apparent *K*_D_ values are identical to those obtained for wild‐type P dimers (Table 1, No. 4 and Figure S10).

### HBGA binding revisited

**Figure 5:**
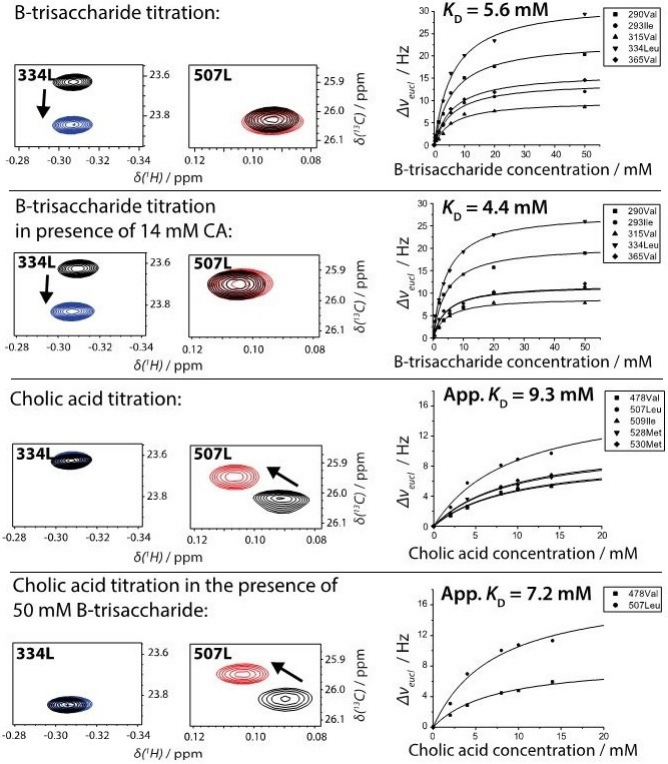
Examples of CSPs in methyl TROSY spectra of GII.4 Saga P dimers in the presence of blood group B‐trisaccharide (B‐Tri) and cholic acid. Left column: CSP of the cross‐peak of the ^13^C methyl group of Leu334 indicates binding of B‐Tri and is only marginally affected by the presence of saturating amounts of CA. Middle column: CSP of the cross‐peak of the ^13^C methyl group of Leu507 indicates binding of CA and is only marginally affected by the presence of saturating amounts of B‐Tri. Right column: Corresponding *K*_D_ values for B‐Tri and apparent *K*_D_ values for CA from binding isotherms using global fitting.

Using CSP titrations we have shown that the affinity of l‐fucose, which constitutes the minimal recognition element of HBGAs,^18^ had been significantly overestimated in preceding studies. We also showed that the same holds true for blood group B‐trisaccharide (B‐Tri) by reevaluating data from a competitive STD NMR experiment.^19^ Therefore, we determined the dissociation constant *K*_D_ for B‐Tri binding to Saga P dimers using a CSP titration based on methyl TROSY experiments before addressing the question whether there is mutual cross talk between HBGA‐binding and bile acid binding. Using a freshly purified sample of NN P dimers we obtained a *K*_D_ value of 5.6 mm (cf. Figure 5), in excellent agreement with the value of 5.5 mm from a competition STD NMR experiment.^10^, ^19^

### Bile acid binding does not affect HBGA binding and vice versa

To answer the question whether bile acid binding and HBGA binding have a mutual impact, we performed methyl TROSY‐based CSP NMR titrations with CA in the presence of saturating amounts of B‐Tri and with B‐Tri in the presence of near‐saturating amounts of CA. The results are summarized in Figure 5 and show that binding affinities of CA and B‐Tri are unaffected by the presence of B‐Tri or CA, respectively.

### Glycyrrhizic acid binds to an adjacent site

To shine some more light on the specificity of the bile acid P‐domain interaction we chose glycyrrhizic acid (GR) as a test compound. GR belongs to the class of saponins, which are amphipathic glycosides, containing a triterpene ring system with structural similarities to the steroid backbone of bile acids. From ^1^H,^15^N TROSY HSQC based CSPs it is clear that GR binds to GII.4 Saga P dimers at a site adjacent to the bile acid site, as this can be seen from Figure S11. Although a comparison with CA binding is impossible, it can be stated that the affinity of GR is of the same order of magnitude as found for CA (Table 1, No. 15).

### Microsecond MD combined with docking reveals a dynamic cavity in the binding region

**Figure 6:**
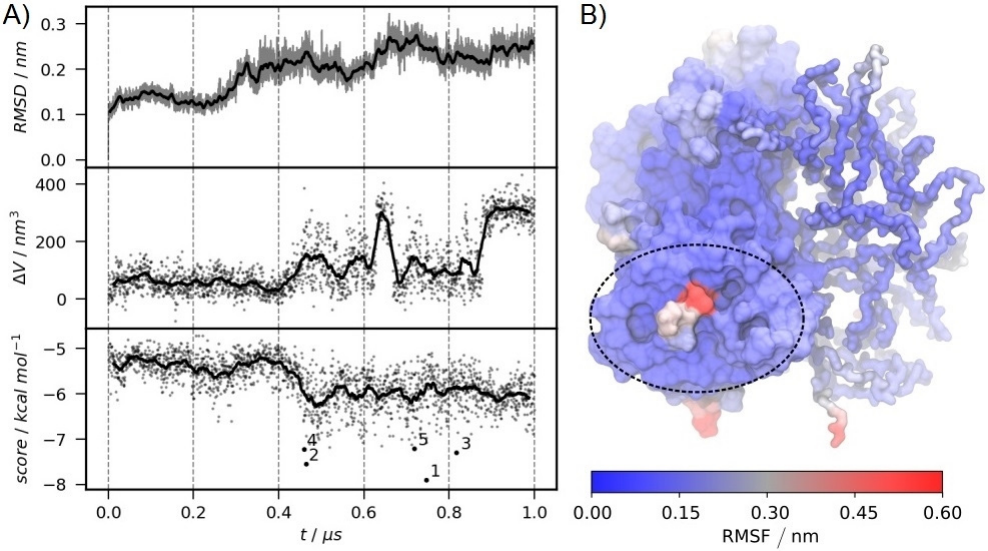
A) Backbone RMSD, difference in pocket volume relative to crystal structure and average bile acid docking score. Solid lines represent moving averages. The shaded area and the dots represent the actual values. The CA ligand poses with the five lowest docking scores were labeled as 1–5 (cf. lowest panel) and were subsequently subjected to MD refinement. B) Backbone RMSF mapped to the protein X‐ray structure (PDB ID: https://www.rcsb.org/structure/4X06). The binding pocket identified by NMR is marked with an ellipsoid.

Crystal structures of GII.4 Saga P dimers (PDB IDs: https://www.rcsb.org/structure/4X06 and https://www.rcsb.org/structure/4OOX) exhibit no accessible binding pocket of sufficient volume to accommodate bile acid molecules. Therefore, the P dimer was subjected to a long all‐atom MD simulation (1 μs), revealing significant dynamics of the backbone as reflected by the root‐mean‐square deviation (RMSD) of backbone atoms in Figure 6 A and the different protein conformations shown in Figure S12. The RMSD relative to the crystal structure fluctuates around 0.10 nm in the first 200 ns simulation time, then increases to 0.20 nm during the next 600 ns, and finally converges to 0.25 nm during the last third of the simulation.

The root‐mean‐square fluctuations (RMSF) of the backbone atoms are used to locate regions of high flexibility. As shown in Figure 6 B the RMSF is especially large at both termini (0.6 nm) and within flexible loop regions (0.3 nm), which can be expected. However, in the crystal structure the C terminus occupies the experimentally identified binding site, preventing bile acid binding. During the MD simulation the binding site volume increases significantly relative to the crystal structure (Figures 6 A and S13). Volume fluctuations between 20 and 130 nm^3^ during the first 400 ns of the simulation can be assigned to small conformational changes of mainly amino acid side chains. After 400 ns, fluctuations increase in amplitude and several distinct changes can be detected due to larger structural, that is, backbone rearrangements, especially concerning the C‐terminal residues 527–530.

### Ensemble docking identifies initial binding modes

The high conformational flexibility of the binding site and the associated fluctuations of the pocket volume do not allow unambiguous identification of accurate bile acid binding poses. Therefore, a large ensemble of *N*=2000 protein conformations was generated by systematically extracting protein conformations at intervals of 0.5 ns. These 2000 receptor conformations were subsequently used for docking of CA, DCA, CDCA and GCDCA. The docking scores (arithmetic mean of the docking scores of CA, DCA, CDCA, and GCDCA to GII.4) for the 2000 docked conformations are shown in Figure 6 A (cf. lowest panel) together with corresponding RMSD values and pocket volume differences Δ*V*. The five lowest scores (highest affinities) are found only after long simulation times of 747.0, 464.5, 817.5, 460.0, and 718.5 ns, with average docking scores of −7.9, −7.6, −7.3, −7.2, and −7.2 kcal mol^−1^, respectively. For these five protein conformations, all of the four bile acid species exhibit lowest docking scores, with GCDCA systematically showing the lowest ones (Figure S14 and Table S3).

It has to be emphasized again, that the best scores were achieved only after 400 ns of MD simulation, correlating with an increase of backbone RMSD values from 0.1 to 0.2 nm. However, the best scoring protein–ligand complexes neither exhibit a distinctly high protein RMSD nor a particularly large pocket volume. Therefore, the use of such a large ensemble of protein conformations is superior to the selection of a small set of conformations via RMSD values or pocket‐shape based clustering as suggested in earlier studies.^20^ Otherwise, important protein conformations critical for ligand binding may remain unresolved.

**Figure 7:**
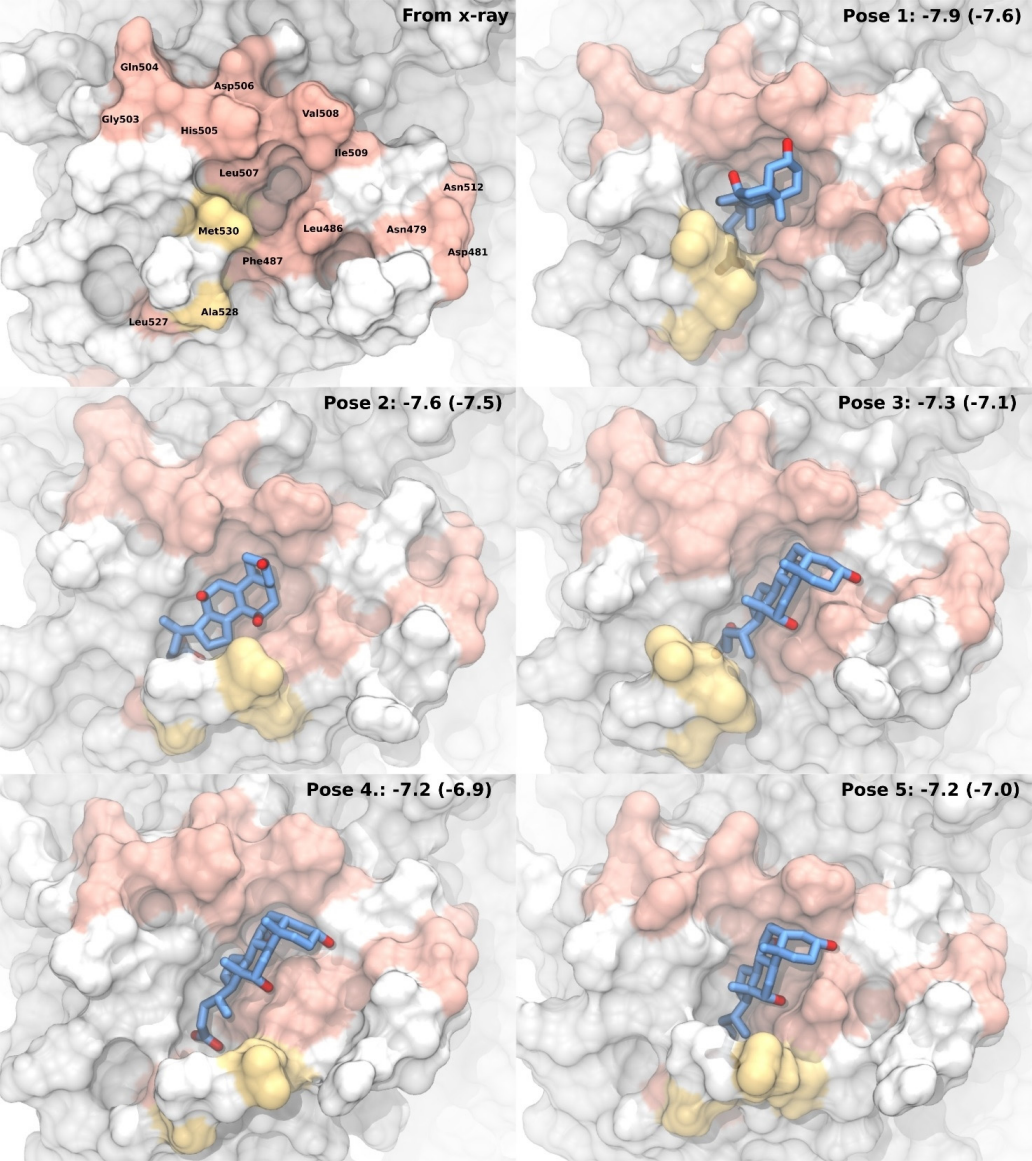
X‐ray structure (in absence of small molecules) and the five top‐scoring poses resulting from dynamic docking of CA to MD snapshots of P dimers. The protein surface is color coded according to experimental CSPs. Backbone CSPs larger than mean+2 *σ* (cf. Figure 1 B) are shown in pale red. CSPs larger than mean+*σ* from methyl TROSY experiments (cf. Figure 4 B) are yellow. CA is shown in blue with oxygen atoms highlighted in red and hydrogen atoms omitted for clarity. The numbers represent the average of all bile acid docking scores with the CA docking score in brackets [kcal mol^−1^].

In the five best‐scoring protein–ligand poses the C‐terminal residues are more solvent exposed as compared with the crystal structure, forming a novel cavity of sufficient size and allowing interactions of the C terminus with the carboxylate groups of the bile acids, as exemplarily shown for CA in Figure 7 (for the other bile acids, see Figure S15). The bile acid orientation is quite similar for all poses with the carboxylate being close to Leu527. In pose 1, the hydroxy groups are facing His505, Leu506 and Val508. In Pose 2, the hydrophilic site is oriented away from the protein, pointing to the solvent. Poses 3–5 are almost identical and feature interactions of the hydroxy groups with protein residues Leu486 and Phe487.

### Equilibrium MD of protein–ligand complexes reflects weak binding with multiple binding modes

**Figure 8:**
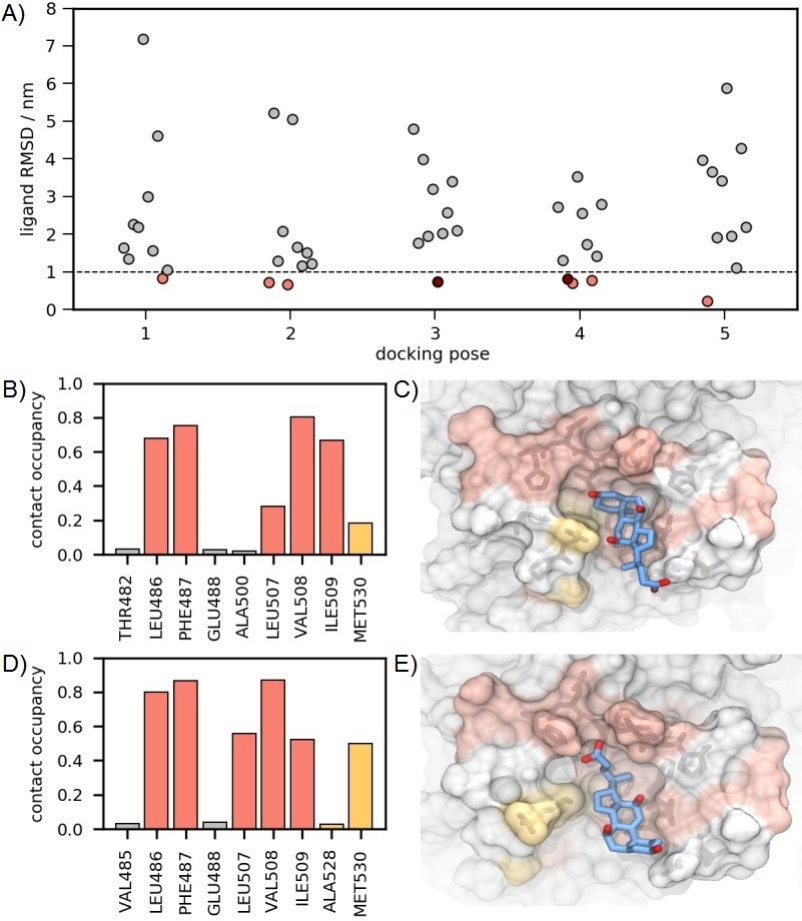
A) MD refinement of docked ligand poses of CA. Each point represents the mean RMSD of the final 10 ns of a 20 ns MD trajectory using the corresponding docking pose as initial coordinates. For each pose 1–5 (cf. Figure 7) ten independent simulations with varying initial velocity distributions were performed. Trajectories with RMSD<1 nm are highlighted as orange or dark red filled circles and were further analyzed for CA–protein contacts. As examples, CA–protein contacts for the red filled circle trajectories are shown in (B) and (D) in detail. B), D) Contact occupancies of CA with backbone nitrogen atoms during the last 10 ns of trajectories Pose 3 Rep 5 (B) and Pose 4 Rep 3 (D). Contact criterion is a distance ≤0.6 nm between the backbone N and at least one heavy atom of CA. Contact amino acids that exhibit significant CSPs are highlighted in red (backbone HSQC) and gold (methyl TROSY), respectively. Proline residues are not considered as they show no NMR signals. Only amino acids with an occupancy >0.02 are shown. C), E) Representative snapshots of stable protein–CA complexes for Pose 3 Rep 5 (C) and Pose 4 Rep 3 (E).

As the poses from docking only approximate true binding modes, we used them as initial configurations for subsequent MD refinement simulations. For each of the five top‐ranked poses of each bile acid molecule, ten independent MD runs of 20 ns each with different initial velocity distributions were performed. The average ligand RMSDs of the 50 MD runs were used as a criterion for the receptor–ligand complex stability (for the full ligand RMSDs, see Figure S16). Here, RMSD>1.0 nm indicates dissociation of the complex, a value between 0.3 and 1.0 nm corresponds to a stable complex with a ligand orientation rearrangement relative to the docked structure, and a RMSD<0.3 nm belongs to a stable binding pose with minor refinement during the MD simulation. Depending on the initial coordinates and velocity distributions, the ligand RMSDs range from 0.2 to 7.1 nm as shown in Figure 8 A. Taking into account the low affinity of CA and the nonideal, approximate initial configuration from docking, the fraction of replicates with stable complexes is as low as 10–30 %. Nevertheless, for each docking pose, at least one trajectory can be found that converged into a stable binding pose of the ligand in the new protein pocket (RMSD<1). These are Pose 1: Replicate (Rep) 8, Pose 2: Rep 4, Pose 2: Rep 0, Pose 3: Rep 5, Pose 4: Rep 3, Pose 4: Rep 7, Pose 4: Rep 2, and Pose 5: Rep 1 (cf. Figure 8 A).

For the stable protein–ligand complex trajectories, the heavy‐atom contact occupancies of CA with the nitrogen atoms of the protein backbone were computed. It is remarkable that Leu486, Phe487, Leu507, Val508 and Ile509 and Met530 exhibit persistent CA contacts in all of the trajectories (Figure S17). This observation is in excellent agreement with the NMR experiments, as the abovementioned amino acids also show significant CSPs. However, some trajectories show persistent contacts with amino acids exhibiting no CSPs, indicating rather short‐lived transient states, possibly undetectable by NMR. MD trajectories Pose 3: Rep 5 and Pose 4: Rep 2 are most consistent with the NMR results and, therefore, are considered most realistic. Both show persistent contacts with the six abovementioned amino acids (see Figure 8 B, D) and almost no contacts to others residues. In the ligand orientations in the stable complexes the hydrophilic side of CA is pointing toward the solvent phase, which is consistent with the experimental epitope mapping (Figure 2). In these poses, CA extends from His505 to Arg484 without making significant contact with the backbone atoms of these amino acids (Figure 8 C, E). The side chains, however, of both residues His505 and Arg484 are positively charged and thus suitable interaction partners for the hydroxy and carboxylate oxygen atoms of CA. The interaction of CA with Leu527 identified from ligand docking cannot be reproduced in the MD simulations. It can be speculated, that the C‐terminal residues 527–530 and CA compete for the binding site, leading to a dynamic binding equilibrium with multiple, transient binding modes. This is experimentally reflected by the broad spatial distribution of residues with significant CSP and the low affinity in the millimolar range of CA binding.

## Discussion

Protein‐based CSP NMR experiments combined with ligand‐based NMR experiments and long‐timescale all‐atom MD simulations have provided a detailed picture of low‐affinity binding of bile acids to NoV capsid protein. For the translation of experimental CSPs into docking models^21^ it was crucial to engage long MD simulations because the low‐affinity bile acid binding site cannot be represented by a single conformation. For instance, in the crystal structure the C terminus blocks access to the binding pocket. Analogous to results of a recent study of the renin binding site,^22^ the discovered low‐affinity site is of high plasticity and allows several modes of binding. MD refinement of initial docking models from dynamic docking (Figure 7) yielded stable docking poses as shown in Figure 8. For these two docking poses qualitative agreement between experimental CSPs and calculated contact occupancies is excellent. We note, however, that a quantitative interpretation is limited as CSPs reflect changes in the magnetic environment of a given nucleus, depending on many parameters such as ring currents of nearby aromatic side chains, electric fields, possible hydrogen bonds, and magnetic anisotropies, preventing a simple correlation with interatomic distances. Therefore, high contact occupancy does not necessarily translate into large CSPs and vice versa. The picture emerging from our studies is a highly dynamic low‐affinity bile acid binding pocket, accommodating bile acids in different orientations (e.g., Figure 8 C, E).

The computational workflow highlights the large uncertainty of automated docking approaches for the identification of binding modes of low affinity binders such as bile acids to shallow, dynamic binding pockets. Even the availability of experimental NMR restraints did not significantly improve the situation. The binding site needs to be thoroughly sampled either by long MD simulations on the microsecond timescale or by enhanced sampling methods. A recent systematic study about conformation selection for docking^23^ pointed out that the true binding mode does not necessarily correlate with pocket volume and shape or clustered conformations. Rather, the conformational ensemble should be sufficiently large and not limited to few cluster representatives, and the docking algorithm should take into account side chain flexibility, as this is nicely supported by our results.

Our results highlight that good docking scores do not necessarily lead to stable protein–ligand complexes in MD trajectories during further refinement as this has been discussed by others recently.^24^ Depending on ligand affinity, many replicates may be necessary to obtain stable complexes with structural integrity and convergence, even after very careful equilibration. Finally, a converged complex with a low RMSD does not necessarily represent an accurate binding mode but can also appear due to improbable or overly strong interactions in the initial configuration. Our study shows once more that careful inspection and rationalization of the computational predictions by comparison with experimental restraints is vital for generating a realistic binding model. Following these arguments, our approach revealed a binding pocket of sufficient volume to accommodate different bile acids, thus suggesting dynamic binding with multiple transient modes that all share backbone contacts with Leu486, Phe487, Leu507, Val508 and Ile509, as well as ligand orientations with the bile acid methyl groups being buried.

Our studies suggest that low‐affinity binding of bile acids is a common feature of NoV capsid proteins independent of genogroup or genotype (Table 1). Dissociation constants are in the low millimolar range and are thus similar to affinities for HBGAs. For GII.4 Saga, an almost complete assignment of backbone NH signals of the P‐domain of the VP1 capsid protein is available, allowing location of the bile acid binding site from specific CSPs. We hypothesize that this binding site is also present in the other NoV strains where we have observed low‐affinity bile acid binding. This hypothesis is supported by the fact that for GII.4 MI001, where we have some tentative backbone NH assignments available, the same region seems to be affected. We are currently working on the backbone and ^13^C‐metyhl assignments of other NoV strains to further substantiate this hypothesis.

Importantly, for GII.4 Saga P dimers no CSPs have been observed at sites corresponding to two other bile acid binding sites described before.4a, 4c As replication of GII.4 NoVs in human intestinal enteroids has been reported to be significantly enhanced by the presence of bile acids,4f and because we can exclude binding to one of the putative high‐affinity sites, we suggest that the low‐affinity interaction with viral capsids provides the molecular basis for understanding the role of bile acids in promoting infection.

To test the possibility that there is mutual cross‐talk between HBGA and bile acid binding we performed NMR binding experiments in the absence and presence of the respective other ligand (Figure 5). Our experiments show that binding of bile acids to the low‐affinity site and binding of HBGAs to the HBGA site are independent events. Likewise, we have shown that the influence of spontaneous deamidation in the HBGA binding site^10^ on the binding affinity for bile acids is negligible. These findings suggest that there is no or very minor cross‐talk between these binding events.

For GII.10 Vietnam026 we expected affinities for bile acids in the micromolar range due to binding to a high‐affinity bile acid binding site adjacent to the HBGA site as described recently.4a In this case we have no isotope‐labeled P dimers available yet, but we have performed STD NMR experiments with GII.10 Vietnam026 VLPs. Unexpectedly, binding isotherms from STD NMR titrations only reflect low‐affinity binding, at least showing that the low‐affinity binding site is present. The failure of observing STD effects resulting from binding to the high‐affinity site is likely due to low rates of dissociation of bile acids from that site, leading to weak or no saturation transfer. Therefore, we conclude that this high‐affinity site is invisible to STD NMR experiments. We are currently working on labeling GII.10 Vietnam026 P‐domains with stable isotopes to further explore this binding site4a with CSP NMR experiments.

In this respect it is of note that the low‐affinity bile acid site is located close to the C terminus of NoV P‐domains. In our studies we have used C‐terminally truncated P‐domains of the VP1 capsid protein because the presence of the highly conserved so‐called arginine tail^25^ leads to aggregation and significantly impedes NMR experiments. On the other hand, all VLP preparations contain the arginine tail. Therefore, if the arginine tail played a role in bile acid binding this should be reflected by different affinities of bile acids for P dimers versus VLPs. From CSP NMR experiments the relative binding affinities of CA binding to Saga P dimers or to Saga VLPs are only about a factor of two apart (Table 1). Such small differences might well be due to slightly different experimental setups and likely do not indicate involvement of the arginine tail in binding to bile acids. In fact, the temperatures for the two types of experiments, CSP versus STD NMR, were different and may explain the observed small alterations. On the other hand, as discussed above for the high‐affinity site present in GII.10 Vietnam026 P‐domains one can speculate that the arginine tail induces conformations that bind to bile acids with higher affinity. This would be invisible to STD NMR and would require CSP NMR experiments using either VLPs or P‐domains including the arginine tail. Neither stable isotope labeling of VLPs nor preparing non‐aggregating samples of P‐domains including the arginine tail is a trivial task. We are currently working on these problems in our laboratory.

At present, we have no complete backbone NH assignments for P dimers of NoV strains other than GII.4 Saga. Therefore, we cannot yet directly identify bile acid binding pockets of these strains from CSP NMR experiments. However, the observation of similar binding affinities of various NoV strains (Table 1) indirectly suggests similar binding modes and sites in all cases. A structure based sequence alignment of NoV VP1 domains available from our previous study^10^ shows that within a given genotype most amino acid positions are highly conserved. Therefore, it is not surprising that some of the amino acids identified to be affected by bile acid binding are also highly conserved. For instance, the stretch of amino acids from R484 to K490 is almost identical for all GII.4 sequences (>98 % sequence identity). In general, the complete C terminus is highly conserved with an average sequence identity of >95 %. Therefore, a comparison among different genogroups and genotypes is more informative. Evaluation of the VP1 C‐terminal sequences of the NoV strains studied in this work demonstrates that mutations of amino acids affected by bile acid binding are possible without impeding binding (cf. Figure S18). This observation matches the results from MD simulations, showing considerable plasticity of the low‐affinity bile acid binding site and thus allowing different poses of bile acids (Figure 8 C, E). It appears that this low‐affinity binding site is rather promiscuous, able to accommodate various bile acids with similar affinities. It will be interesting to further scrutinize the consequences of our findings in cell culture systems and animal models.

## Conclusion

This study highlights the complex role of bile acids in norovirus infection from a structural point of view and underlines the potential of protein‐ and ligand‐based NMR binding experiments in combination with long‐timescale MD simulations to portrait low‐affinity binding events. A low‐affinity bile acid binding site appears to be a common feature of a variety of human NoV strains. As affinities in the millimolar range match concentrations of bile acids in the intestine,^26^ low‐affinity bile acid binding should be saturated when the virus is residing in the intestine. It would be very interesting to find NoV strains lacking the ability of binding bile acids at the C terminus and to study the consequences for infection. We speculate that “soft recognition” of bile acids affects the stability of viral capsids and in turn modulates infection. In the light of the development of novel and better accessible NoV cell culture systems^27^ this hypothesis seems to be verifiable in the near future.

## Experimental Section

**Protein biosynthesis and purification**: Non‐deamidated [U‐^2^H,^15^N] GII.4 Saga 2006 (GenBank accession number BAG70518.1, residues 225–530) and GII.4 MI001 (GenBank accession number AGQ46694.1, residues 225–530) P‐domains were synthesized and purified as described previously.^10^ The N373D mutant of the GII.4 Saga 2006 protruding domain was generated by site‐directed mutagenesis (Promega). ^13^C‐methyl (U‐[^2^H], MIL^ProS^V^ProS^A)‐labeled, non‐deamidated GII.4 Saga 2006 and GII.17 Kawasaki308 P domains (residues 225–530, GenBank accession number BAR42289) were expressed according to a modified version of the above‐mentioned protocol as given in the Supporting Information. Briefly, a solution containing isotopically labeled precursors (for details see Table S4) was added after cell adaptation to D_2_O medium when OD_600_ had reached a value of 0.6–0.8. The culture was incubated at 16 °C for 1 h, and protein overexpression was induced with 1 mm isopropyl β‐d‐1‐thiogalactopyranoside (IPTG). Growth was continued at 16 °C until the stationary phase was reached. Cells were harvested by centrifugation at 5000 *g* for 20 min at 4 °C. Deamidated GII.4 Saga 2006 P‐domains were obtained by ion‐exchange chromatography from a sample incubated for 20 days at 25 °C.

GI.1 Norwalk, GII.4 Saga4/2006, GII.10 Vietnam026, GII.17 Kawasaki308 and GII.17 Saitama/T87 VLPs (GenBank accession numbers AMD33538.1, BAG70518.1, AAT12445.1, BAR42289.1 and AII73747.1) were donated by Dr. Grant Hansman (University of Heidelberg and DKFZ, Germany). GII.7 RKI VLPs (GenBank accession number AGQ57036.1) were a gift from Prof. Stefan Taube (University of Lübeck, Germany). GII.4 Ast6139 VLPs (GenBank accession number CAE47529.1) were donated by Prof. Francisco Parra (University of Oviedo, Spain).

**Bile acids and other ligands**: Cholic acid (CA), glycochenodeoxycholic acid (GCDCA), sodium taurochenodeoxycholate (TCDCA), deoxycholic acid (DCA), chenodeoxycholic acid (CDCA), taurocholic acid (TCA), sodium glycocholate hydrate (GCA) and glycyrrhizic acid (GR) were purchased from Sigma–Aldrich. Blood group B‐trisaccharide α‐l‐Fuc‐(1,2)‐[β‐d‐Gal‐(1,3)‐]‐β‐d‐Gal‐(1,N)‐N_3_ was a gift from Dr. Hanne Peters in our laboratory and had been obtained via enzymatic synthesis from (α‐l‐Fuc‐(1,2)‐β‐d‐Gal‐(1,N)‐N_3_, which was a gift from Prof. Javier Pérez‐Castells (CEU San Pablo, Madrid, Spain).

**NMR spectroscopy**: STD NMR experiments were acquired at 277 K on a Bruker Avance III HD 600 MHz NMR spectrometer equipped with a TXI room temperature probe. All other NMR experiments were recorded on a Bruker AV III 500 MHz NMR spectrometer equipped with a TCI cryogenic probe at 298 K if not specified otherwise. Data were processed with TopSpin 3.6, and peak positions were extracted using CCP NMR 2.4.2.^28^ A backbone assignment is available for Saga GII.4 P dimers and is deposited with the BioMagResBank with the accession code 27445.

**Backbone and side‐chain chemical shift perturbation experiments**: ^1^H,^15^N TROSY HSQC spectra were acquired with 8–24 scans with 2048 data points in *t*_2_ and 430 increments in the indirect dimension *t*_1_. The acquisition time was 128 ms in *t*_2_ and 121 ms in *t*_1_. The relaxation delay was set at 1.5 s. The NMR samples contained 100–120 μm [U‐^2^H,^15^N] Saga 2006 or MI001 P dimers, 200 μm [D_6_]DSS, 300 μm imidazole, 100 mm NaCl, 75 mm sodium phosphate buffer (pH 7.3), 0.02 % NaN_3_ and 10 % D_2_O. CA has been titrated up to final concentrations of 15 mm (Saga 2006) and 18 mm (MI001). GR has been titrated up to 25 mm, GCDCA up to 15 mm. Other bile acids have only been added with a single concentration of 2 mm. Bile acid titration stocks were prepared with final concentrations up to 300 mm in 75 mm sodium phosphate buffer, 100 mm NaCl (pH 7.3). The pH of the titration stocks was increased by titration of NaOH until all solid dissolved and subsequently re‐adjusted to pH 7.3. Imidazole signals were used as internal standard^29^ to monitor the pH during titrations as described previously (see supplementary data section 2.3 of ref. 10).

Methyl TROSY spectra^30^ were acquired with 4–48 scans with 1024 data points in *t*_2_ and 512 increments in the indirect dimension (*t*_1_). The acquisition time was 137 ms in *t*_2_ and 120 ms in *t*_1_. The relaxation delay was set at 1.5 s. The NMR samples contained 16.5–31 μm of MIL^ProS^V^ProS^A‐labeled GII.4 Saga or GII.17 Kawasaki308 P dimers in 75 mm sodium phosphate buffer (pH 7.4) in D_2_O (>99 %). The deamidation status of individual P dimer samples depended on the storage time and is described in the main text. Samples contained 100 mm NaCl, 100 μm [D_6_]DSS, 100 μm imidazole and 0.02 % NaN_3_. Samples were titrated to a final concentration of CA of 14 or 20 mm. Bile acid titrations were performed as described above, with the only difference that NaOD in D_2_O was used to prepare bile acid titration stocks with a final pH 7.4.

**Saturation transfer difference (STD) NMR**: A train of 50 ms Gaussian‐shaped radio frequency pulses separated by 1 ms for a total duration of 2 s were used for protein irradiation.^31^ For extracting *K*_D_ values from STD initial growth rates, 0.25, 0.5, 0.75, 1, and 2 s saturation times were used. In all cases, an attenuation of 40 dB was chosen resulting in a 680.5° flip angle. The water signal was suppressed using excitation sculpting,^32^ and for protein signals of P dimers were attenuated applying a 20 ms spinlock filter before acquisition. The acquisition time was set at 1.96 s with an additional relaxation delay of 5–20 s. On and off resonances were set at −4 ppm and 200 ppm, respectively.^33^ The number of scans ranged from 200 to 2400.

Samples containing VLPs were prepared at 0.41 to 1 mg mL^−1^ VP1 concentration (6.9 to 17.7 μm binding sites) in PBS pH 7.3, 100 μm [D_6_]DSS, 0.02 % NaN_3_ and 10 % D_2_O. Samples were titrated with cholic acid to a final concentration of 15–20 mm, with GCA and TCDCA (1 mm each) or with TCA and GCDCA (1 mm each). Bile acid stock solution were prepared as described above for backbone chemical shift perturbation experiments. For N373D Saga P dimers, one sample containing 45 μm P dimers, 100 μm [D_6_]DSS in 20 mm fully deuterated sodium phosphate buffer (pH 7.45) was prepared and titrated with CA up to a final concentration of 12 mm.

**Assessing critical micelle concentrations**: Critical micelle concentrations of CA and GCDCA have been estimated from chemical shift perturbations of the C19 methyl group in a series of 1D ^1^H NMR spectra of the bile acids at different concentrations in 75 mm sodium phosphate buffer, 100 mm NaCl (pH 7.3) with 10 % D_2_O.

**Determination of (apparent) dissociation constants** ***K***_**D**_: Dissociation constants *K*_D_ were calculated either from CSPs, STD‐AF or STD‐AF_0_ by using Equation (1):

(1) $$O=\frac{K_{D}+{\left[L\right]}_{T}+{\left[P\right]}_{T}-\sqrt{{\left(K_{D}+{\left[L\right]}_{T}+{\left[P\right]}_{T}\right)}^{2}-4{\left[L\right]}_{T}{\left[P\right]}_{T}}}{2{\left[P\right]}_{T}}O_{max}$$

in which *O* is the experimentally observed CSP, STD‐AF, or STD‐AF_0_ at each ligand concentration. [P]_T_ and [L]_T_ are the total protein and ligand concentrations, respectively, and *O*_max_ is the observable at saturation with ligand.

For the experiments based on backbone and side‐chain chemical‐shift perturbations, CSPs were calculated as Euclidean distances Δ*ν*_Eucl_ in Hz according to Equation (2):^34^

(2) $$\Delta v_{Eucl}\;\left(Hz\right)=\sqrt{{\Delta v_{H}}^{2}+{\Delta v_{X}}^{2}}$$

for which *X* is either ^15^N or ^13^C, Δ*ν*_H_ and Δ*ν*_X_ the CSPs of ^1^H and ^15^N or ^13^C resonances, respectively, at a given ligand concentration. Global nonlinear least‐squares fitting of Eq. (1) to CSPs (mean+2*σ* for ^1^H,^15^N TROSY HSQC and mean+*σ* for methyl TROSY experiments) furnished dissociation constants *K*_D_.

To derive *K*_D_ values from STD NMR titrations, STD amplification factors (AF) were calculated by using Equation (3):^15^

(3) $$\mathrm{STD}\text{-}\mathrm{AF}=\frac{I{}_{0}-I{}_{\mathrm{sat}}}{I{}_{0}}\times \mathrm{ligand}\;\mathrm{excess}$$

where *I*_0_ and *I*_sat_ are the signal intensities in the off‐ and on‐resonance spectra, respectively. Ligand excess refers to the ratio of ligand concentration over protein concentration. STD‐AF values were plotted against cholic acid concentration. Fitting Equation (1) to the data delivered dissociation constants *K*_D_.

Calculation of *K*_D_ values from STD initial growth rates,^16^ STD‐AF values were measured as a function of the saturation time *t*_sat_ and fitted to Equation (4):

(4) $$\mathrm{STD}\text{-}\mathrm{AF}\left(t{}_{\mathrm{sat}}\right)=\mathrm{STD}\text{-}\mathrm{AF}{}_{\max}\left[1-\exp\left(-k{}_{\mathrm{sat}}t{}_{\mathrm{sat}}\right)\right]$$

with STD‐AF_max_ being the maximum STD‐AF for a given proton, *k*_sat_ the saturation rate constant, and *t*_sat_ the saturation time. Initial slopes STD‐AF_0_ were obtained from STD‐AF_0_=STD‐AF_max_ *k*_sat_, and were plotted as a function of the ligand concentration. Fitting Equation (1) to the resulting binding isotherms delivered dissociation constants *K*_D_. For curve fitting Origin2016G (OriginLab) was used.

**Protein structural models**: The protein model was generated from RCSB PDB ID: https://www.rcsb.org/structure/4OOX 35 using the CHARMM‐GUI PDB reader.^36^ Histidine moieties were protonated according to possible hydrogen bond formations with neighboring amino acids. In particular, histidine residues 292, 347, 417, 460, and 501 were protonated at the Nϵ position, histidines 378, 396, 414, and 490 at the Nδ. For histidine 505 we chose to protonate at both Nϵ and Nδ, as His505 shows such a high CSP that a strong electrostatic interaction was assumed. Because the protein is a homodimer with two identical subunits, we explicitly note that both units were modeled in order to fully account for protein–protein interactions and possible allostery. The protein termini were assumed to be charged (R‐NH_3_^+^, R‐COO^−^). The models for the bile acids were generated using the CHARMM‐GUI ligand reader and modeler^37^ with the coordinates from RCSB ligand Expo.^38^ CHARMM force field parameters^39^ were used in all cases.

Preparation for the initial protein MD simulation model and generation of MD input files was done with the CHARMM‐GUI quick MD simulator.^40^ We note that the analyzed all‐atom microsecond MD trajectory belongs to the set of long co‐solvent simulations, in which we added five molecules of CA to the previously generated model (randomly distributed and corresponding to a concentration of 10 mm). In this trajectory we recorded neither long‐lasting protein–CA contacts, nor CA aggregation. Preparation of the before mentioned structures and the protein–ligand complex simulations as well as the conduction of all of the MD simulations was achieved with GROMACS and GROMACS tools ver. 5.1.5.^41^ All models had a cubic simulation box with at least 2 nm distances from the protein in every direction and were solvated with TIP3P^42^ water, ionized to 0.15 m NaCl.

**Simulation protocol**: All simulations were performed with a standard protocol consisting of 5000 steps steepest descent minimization, 100 000 steps NVT equilibration, 100 000 steps NPT equilibration and production (1000 ns for protein without bound ligands, 20 ns for protein–ligand complexes) in the NPT ensemble. For all MD steps, a time step of 0.002 ps was used and a Verlet cutoff scheme^43^ was applied. Temperature coupling was achieved with the Nosé–Hoover method^44^ (target temperature of 303.15 K, coupling constant of 0.4 ps during equilibration and 2.0 ps during production). Protein (plus the bile acid ligand, if present) and solvent (including water and ions) were coupled individually. The initial temperature distributions were generated according to a Maxwell–Boltzmann distribution at 293.15 K. Pressure coupling was achieved by the Berendsen barostat^45^ during equilibration and by Parinello–Rahman^46^ during production (both using a coupling constant of 2 ps and a reference pressure of 1 bar). Protein and ligand heavy atoms were restrained during equilibration. Hydrogen bonds were constrained, with constraints solved by LINCS.^47^ The nonbonded interaction cutoff was 1.2 nm. Electrostatics were computed with the PME method.^48^

**Analysis of trajectories**: Backbone RMSDs and RMSFs were calculated using GROMACS tools. Translational and rotational displacements of the entire complex were removed by fitting the trajectory to the crystal structure. The differences in pocket volume was calculated with POVME 3.0.^49^ The search space for cavities was set up manually as shown in Figure S13. For the pocket volume and docking calculation, the trajectory was aligned for the significantly perturbed residues as shown in Figure S12.

The docking scores and poses were computed with AutoDock Vina^50^ (ver. 1.12). Receptor (GII.4 P dimer) and ligands (bile acids CA, DCA, CDCA, and GCDCA) were prepared for docking with AutoDock tools.^51^ The protein was kept rigid, whereas all rotatable bonds of the bile acids were flexible. Gasteiger partial charges^52^ were assigned to receptor and ligand. The cubic search space was set up to encompass all perturbed amino acids in the binding region (Figure S12). The search was performed with an exhaustiveness of 64.

Contact analysis was performed with MDTraj.^53^ For each frame, for each amino acid, a contact was counted if at least one heavy atom of CA was in proximity of 0.6 nm or less to its backbone nitrogen. The contact occupancy of an amino acid is the number of counted contacts dived by the number of frames. Molecule images were rendered with VMD ver. 1.9.3.^54^

## Conflict of interest

*The authors declare no conflict of interest*.

## Supporting information

As a service to our authors and readers, this journal provides supporting information supplied by the authors. Such materials are peer reviewed and may be re‐organized for online delivery, but are not copy‐edited or typeset. Technical support issues arising from supporting information (other than missing files) should be addressed to the authors.

Supplementary
