# Supplementary material for: Chemical‐Shift Perturbations Reflect Bile Acid Binding to Norovirus Coat Protein: Recognition Comes in Different Flavors
Source: Chembiochem. 2019 Dec 5;21(7):1007–21. doi: 10.1002/cbic.201900572 (PMC7186840; doi:10.1002/cbic.201900572)
Supplement: Supplementary file 1 — Supplementary [file CBIC-21-1007-s001.pdf]

CHEMBIOCHEM

Supporting Information

Chemical-Shift Perturbations Reflect Bile Acid Binding to Norovirus Coat Protein: Recognition Comes in Different Flavors

Robert Creutznacher,[a] Eric Schulze,[b] Georg Wallmann,[a] Thomas Peters,*[a] Matthias Stein,[b] and Alvaro Mallagaray[a]

cbic_201900572_sm_miscellaneous_information.pdf

Table of Contents

Fig. S1 - Chemical shift perturbations of [U-^{2}H,^{15}N] labeled GII.4 Saga P-dimers with DCA, GCDCA, and CDCA 2

Fig. S2 - STD NMR spectra of CA, GCA, GCDCA, TCA, and TCDCA in the presence of NoV VLPs 3

Fig. S3 - Binding epitope of CA bound to GII.4 Saga VLPs from STD NMR buildup curves 6

Fig. S4 - Concentration dependence of chemical shifts of GCDCA and CA 6

Fig. S5 and Table S1 - STD NMR titrations with a single saturation time of 2s 7

Fig. S6 and Table S2 - STD NMR titrations of GII.4 Saga VLPs with CA using initial STD growth rates 9

Fig. S7 - Tentative assignments in ^{1}H,^{15}N TROSY HSQC spectra of [U-^{2}H,^{15}N]-labeled MI001 P-dimers 11

Fig. S8 - CSP titration of MIL^{ProS}V^{ProS}A ^{13}C-methyl labeled Kawasaki308 P-dimers with CA (methyl TROSY spectra) 11

Fig. S9 - Binding isotherm from a CSP titration of [U-^{2}H,^{15}N] Saga P-dimers with GCDCA 12

Fig. S10 - STD NMR spectra of CA in the presence of N373D GII.4 Saga P-dimers 13

Fig. S11 - Binding of glycyrrhizin (GR) to GII.4 Saga P-dimers and VLPs: CSPs in a ^{1}H,^{15}N TROSY HSQC spectrum of [U-^{2}H,^{15}N] labeled P-dimers and STD NMR spectrum of CA in the presence of VLPs 14

Fig. S12 - Backbone dynamics of GII.4 Saga P-dimers from MD 15

Fig. S13 - Computation of volume and shape of the binding site 15

Fig S14 - Docking scores of DCA, CDCA, and GCDCA to an ensemble of GII.4 Saga P-dimers conformations 16

Fig. S15 and Table S3- Top five scoring protein-ligand docking poses for CA, DCA, CDCA and GCDCA and docking scores 17

Fig. S16 - Ligand RMSD curves over simulation time of the 50 CA:P-dimers complex MD simulations 18

Fig. S17 - Contact occupancies between CA and backbone nitrogen atoms 19

Table S4 - Protein biosynthesis of ^{13}C-methyl labeled P-dimers 20

Table S5 - Summary of bile acid binding results with different NoV strains 21

Fig. S18 - Structural alignment of NoV sequences 22

Fig. S1 - Chemical shift perturbations of [U-^{2}H,^{15}N] labeled GII.4 Saga P-dimers with DCA, GCDCA, and CDCA

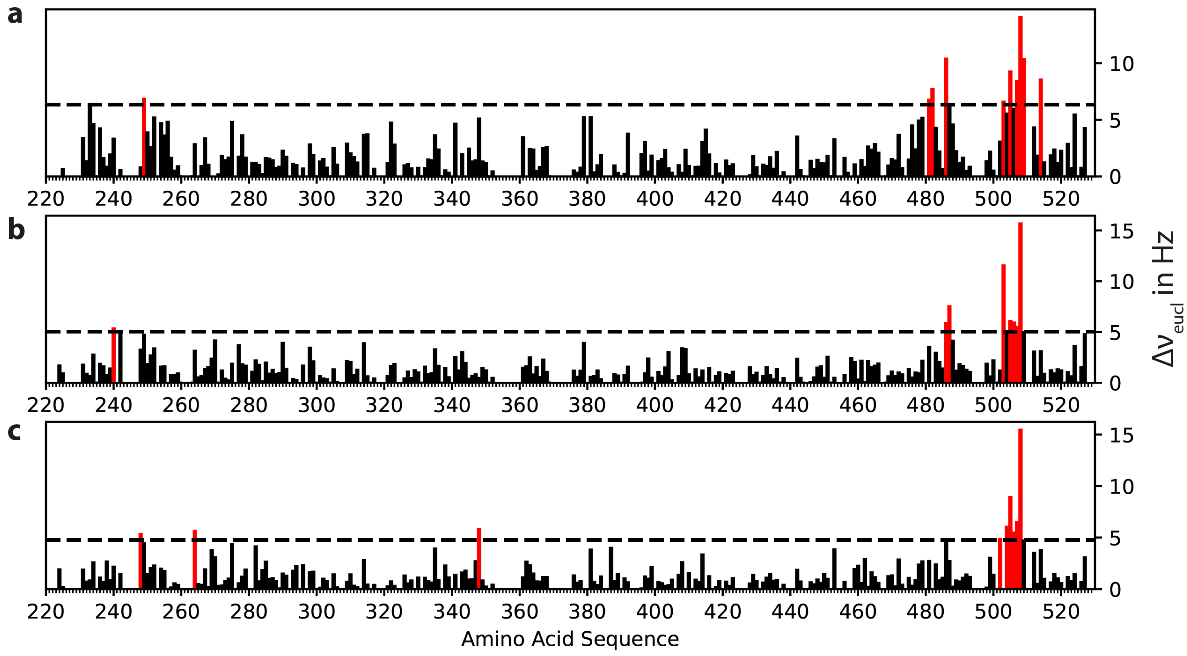

Figure S1: Chemical shift perturbations (CSPs) observed for the interaction of [U-^{2}H,^{15}N] labeled GII.4 Saga norovirus (100 μM) with **a)** DCA, **b)** GCDCA and **c)** CDCA. CSPs larger than *mean+2*σ are indicated in red. All bile acids were dissolved at 2 mM concentration. Experiments were acquired at 500 MHz and 298 K.

Fig. S2 - STD NMR spectra of CA, GCA, GCDCA, TCA, and TCDCA in the presence of NoV VLPs

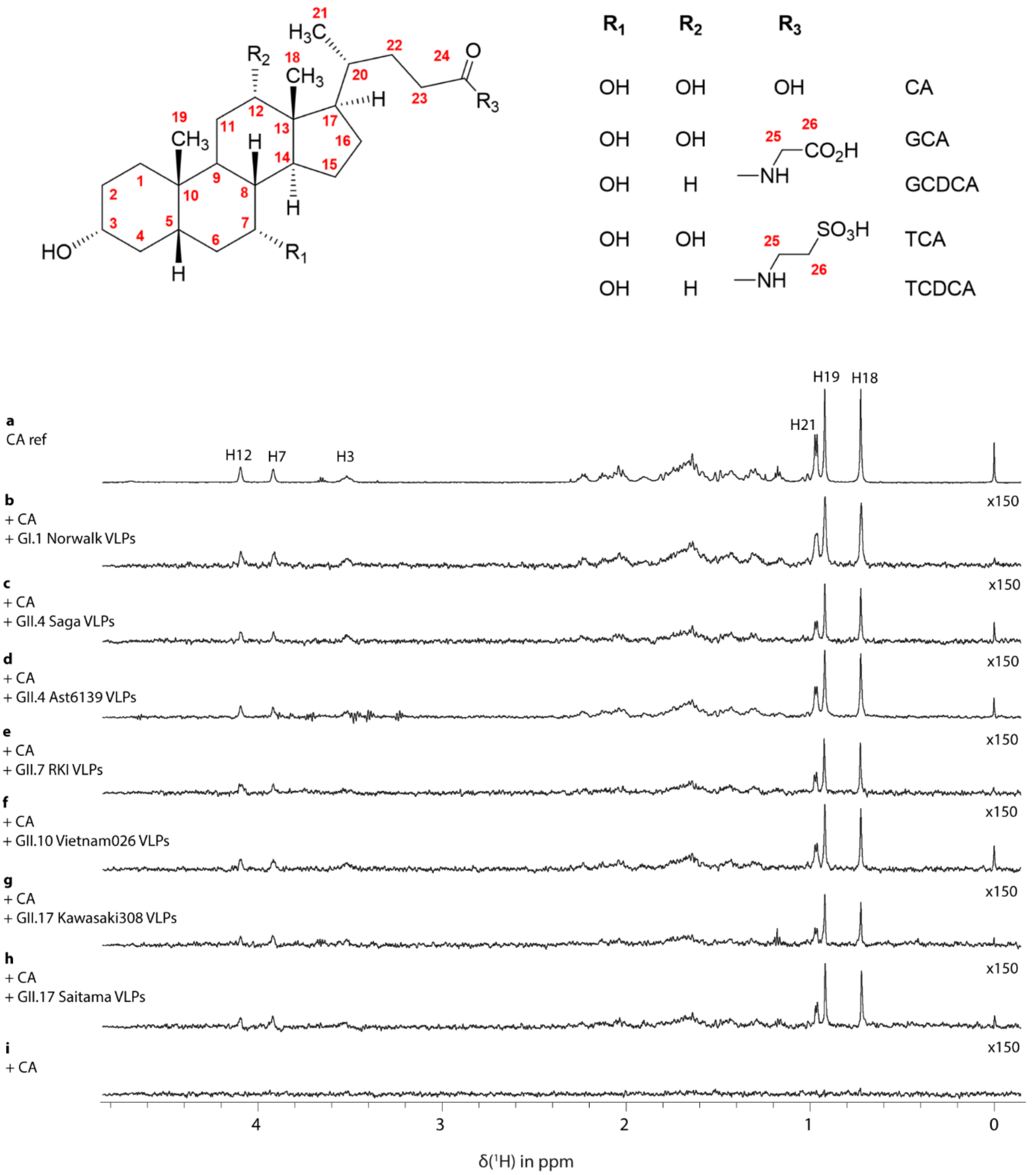

Figure S2: (*Upper panel*) Schematic representation of bile acids used in the experiments with VLPs. (*Lower panel*) STD NMR experiments reflecting the interaction of human NoV VLPs with CA. From top to bottom: **a)** Reference spectrum of CA, **b)** 1 mg/ml VP1 (17.7 µM binding sites (b.s.)) GI.1 Norwalk VLPs, **c)** 1 mg/ml VP1 (16.9 µM b.s.) GII.4 Saga VLPs, **d)** 1 mg/ml VP1 (17 µM b.s.) GII.4 Ast6139 VLPs, **e)** 0.56 mg/ml VP1 (9.4 µM b.s.) GII.7 RKI VLPs, **f)** 0.93 mg/ml VP1 (15.5 µM b.s.) GII.10 Vietnam026 VLPs, **g)** 1 mg/ml VP1 (16.8 µM b.s.) GII.17 Kawasaki308 VLPs, **h)** 1 mg/ml VP1 (17.0 µM b.s.) GII.17 Saitama/T87 VLPs and **i)** no protein. CA was at 1 mM concentration in all the experiments. Spectra were acquired at 600 MHz with the temperature set at 277 K.

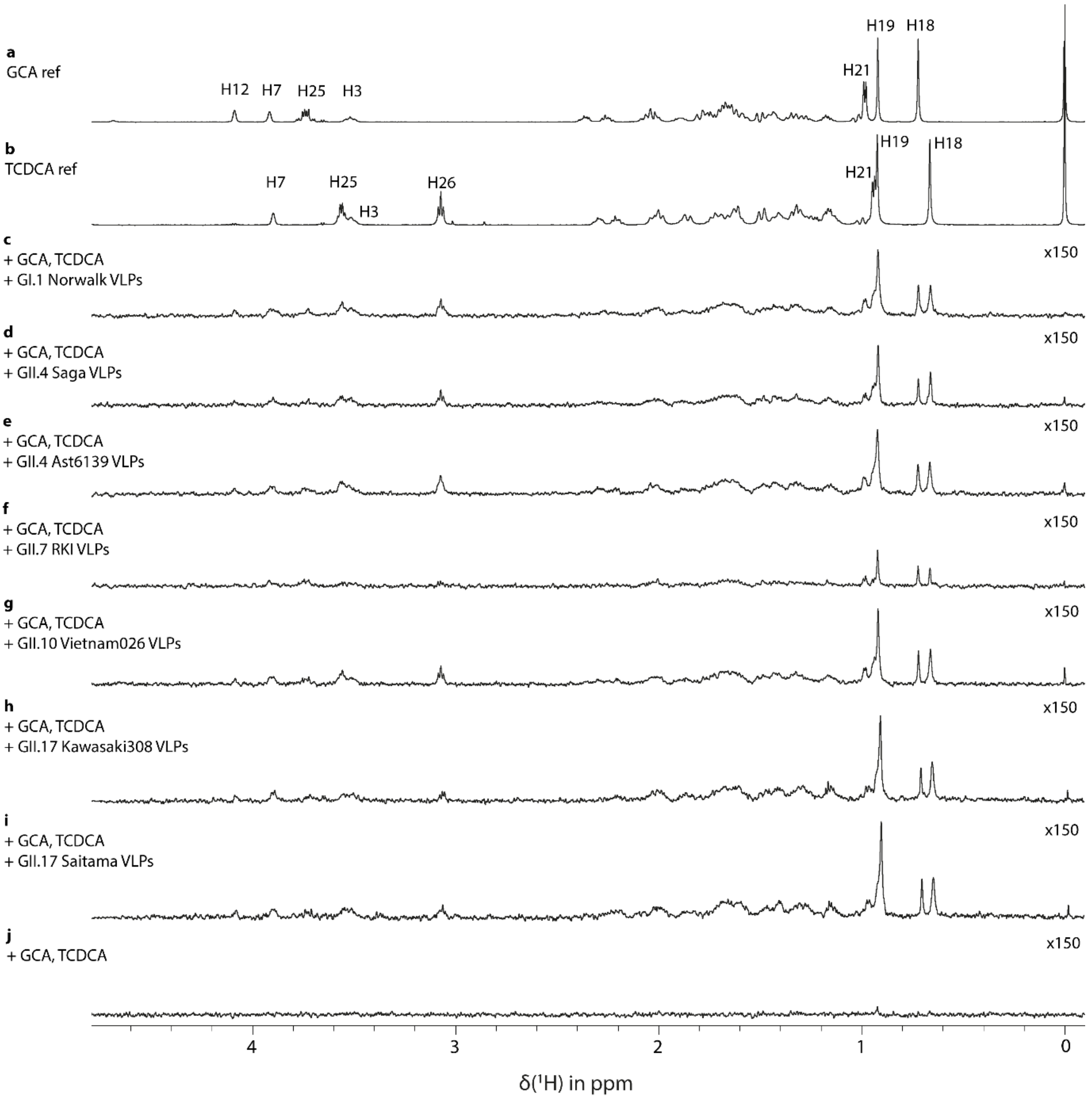

Figure S2 - continued: STD experiments showing the interaction of human NoV VLPs with GCA and TCDCA. From top to bottom: Reference spectrum of **a)** GCA and **b)** TCDCA, **c)** 0.86 mg/ml VP1 (15.2 µM b.s.) GI.1 Norwalk VLPs, **d)** 0.86 mg/ml VP1 (14.5 µM b.s.) GII.4 Saga VLPs, **e)** 0.86 mg/ml VP1 (14.6 µM b.s.) GII.4 Ast6139 VLPs, **f)** 0.41 mg/ml VP1 (6.9 µM b.s.) GII.7 RKI VLPs, **g)** 0.78 mg/ml VP1 (13 µM b.s.) GII.10 Vietnam026 VLPs, **h)** 1 mg/ml VP1 (16.8 µM b.s.) GII.17 Kawasaki308 VLPs, **i)** 1 mg/ml VP1 (17.0 µM b.s.) GII.17 Saitama/T87 VLPs and **j)** no protein. GCA and TCDCA were at 1 mM concentration each in all the experiments. Spectra were acquired at 600 MHz with the temperature set at 277 K.

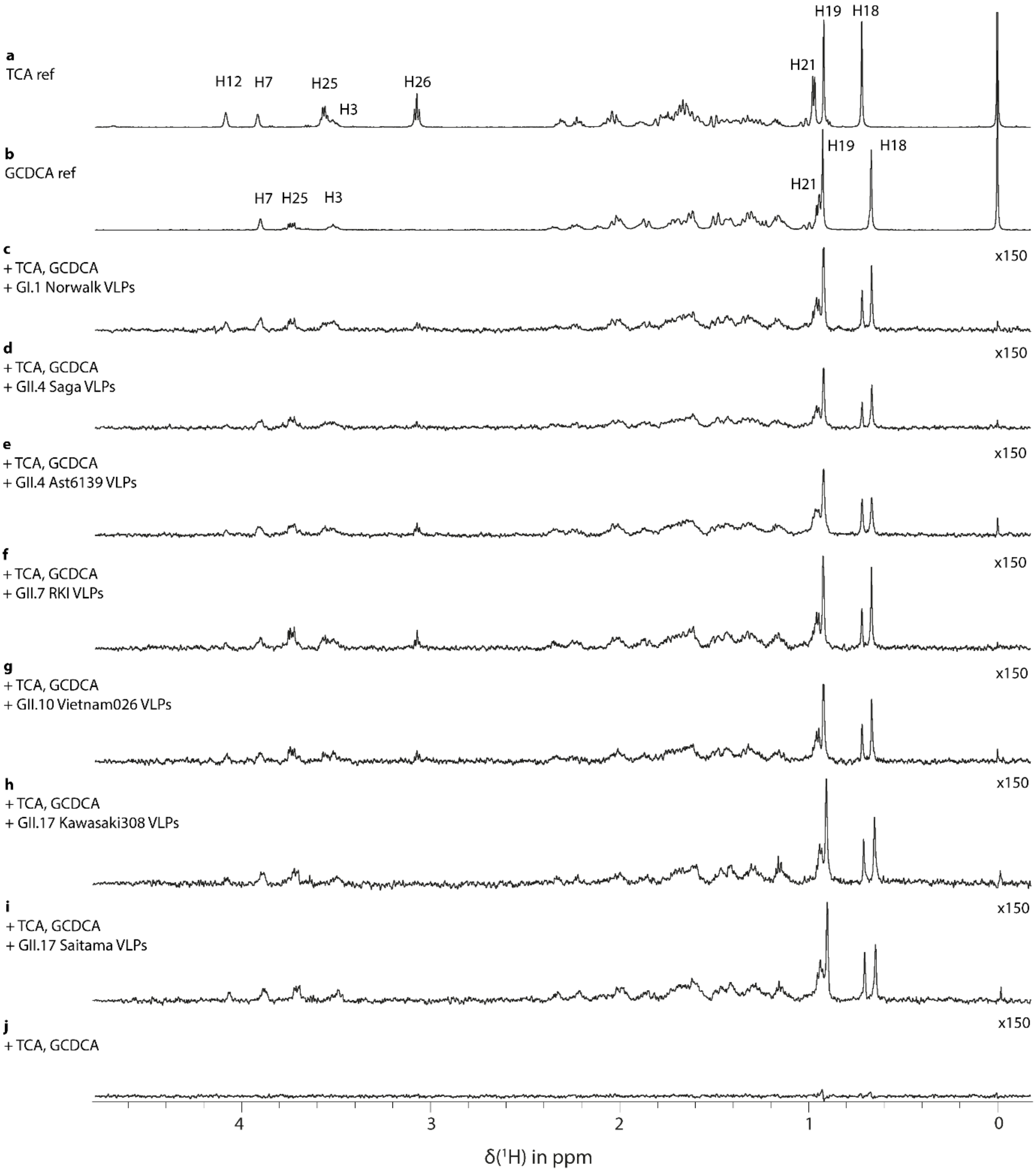

Figure S2 - continued: STD experiments showing the interaction of human NoV VLPs with TCA and GCDCA. From top to bottom: Reference spectrum of **a)** TCA and **b)** GCDCA, **c)** 0.86 mg/ml VP1 (15.2 µM b.s.) GI.1 Norwalk VLPs, **d)** 0.86 mg/ml VP1 (14.5 µM b.s.) GII.4 Saga VLPs, **e)** 0.86 mg/ml VP1 (14.6 µM b.s.) GII.4 Ast6139 VLPs, **f)** 0.41 mg/ml VP1 (6.9 µM b.s.) GII.7 RKI VLPs, **g)** 0.78 mg/ml VP1 (13 µM) GII.10 Vietnam026 VLPs, **h)** 1 mg/ml VP1 (16.8 µM b.s.) GII.17 Kawasaki308 VLPs, **i)** 1 mg/ml VP1 (17.0 µM b.s.) GII.17 Saitama/T87 VLPs and **j)** no protein. TCA and GCDCA were at 1 mM concentration each in all the experiments. Spectra were acquired at 600 MHz with the temperature set at 277 K.

Fig. S3 - Binding epitope of CA bound to GII.4 Saga VLPs from STD NMR buildup curves

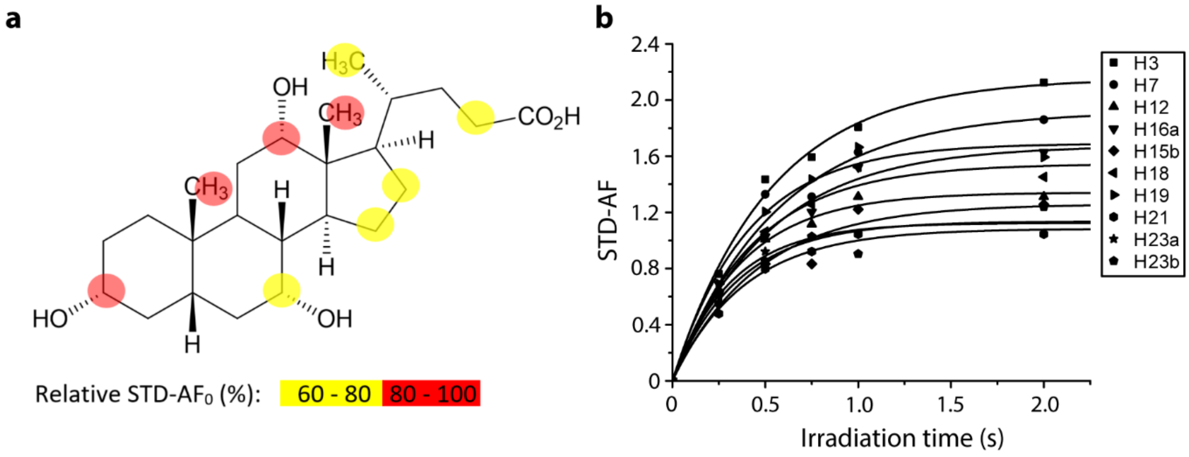

Figure S3: Binding epitope of **a)** CA bound to GII.4 VLPs from **b)** STD NMR buildup curves. STD-AF: STD amplification factors. Experiments were performed at 600 MHz and 277 K.

Fig. S4 - Concentration dependence of chemical shifts of GCDCA and CA

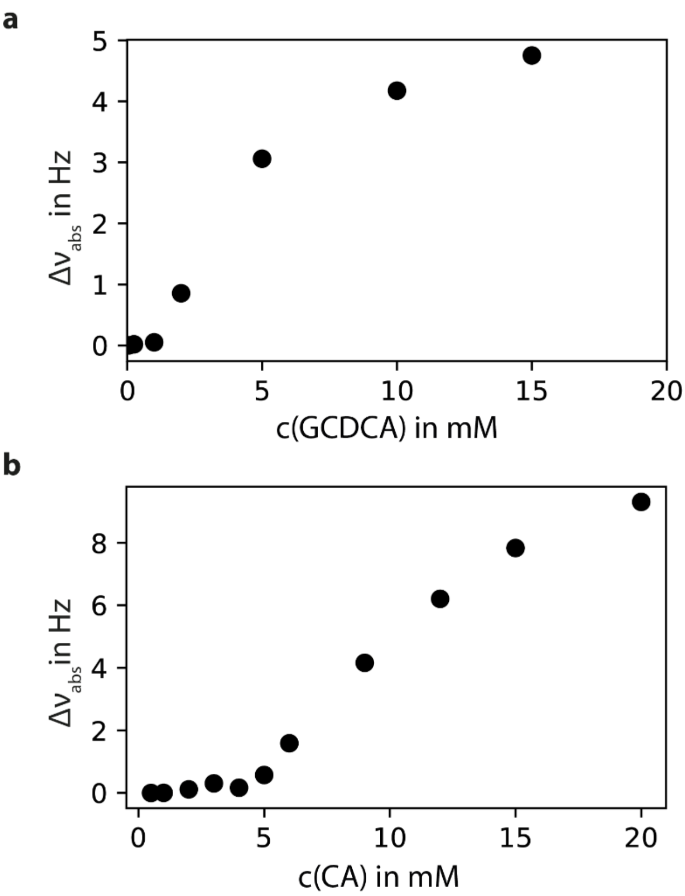

Figure S4: Absolute ^{1}H chemical shift perturbations of **a)** GCDCA and **b)** CA of the representative C19 methyl group signal. Aggregation starts at ~ 1 mM GCDCA and at ~ 4 mM CA. Experiments were performed at 500 MHz and 298 K.

Fig. S5 and Table S1 - STD NMR titrations with a single saturation time of 2s

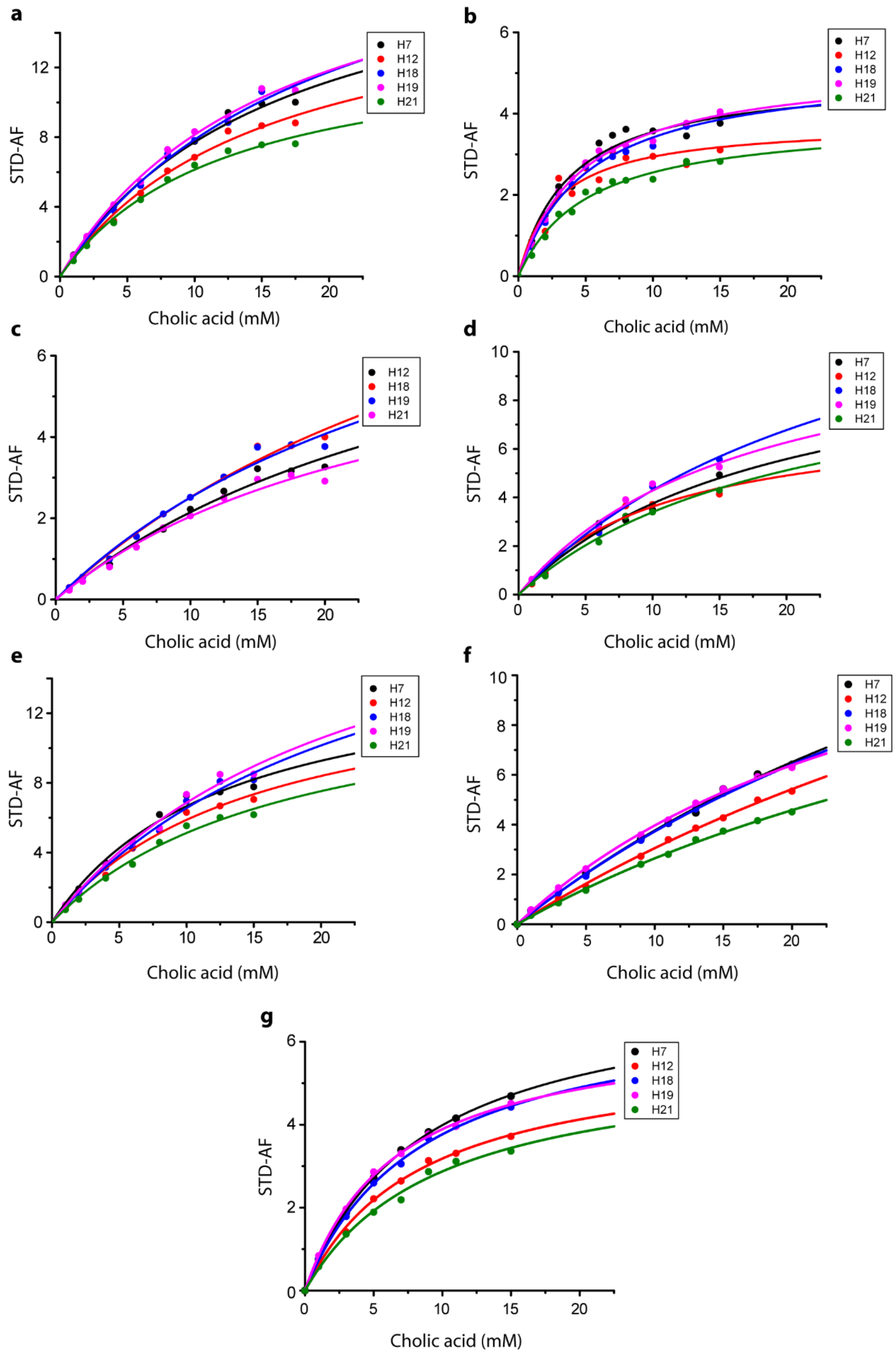

Figure S5: Binding isotherms obtained from STD NMR titrations with CA using single saturation times of 2s. **a)** GI.1 Norwalk VLPs, **b)** GII.4 Saga VLPs, **c)** GII.4 Ast6139 VLPs, **d)** GII.7 RKI VLPs, **e)** GII.10 Vietnam026 VLPs, **f)** GII.17 Kawasaki308 VLPs and **g)** GII.17 Saitama/T87 VLPs. Only isolated signals from CA were selected and fitted to Eq. 1 to deliver dissociation constants K_D (compare Table S1 and Table 1 of the main text). STD NMR experiments were performed at 600 MHz and 277 K.

Table S1: Dissociation constants K_D for individual protons of CA obtained from titration of VLPs with CA from STD NMR titrations using a single saturation time of 2s. The lowest values are highlighted in bold and are assumed to be closest to the apparent K_D. Proton H12 from entry 2 was not used due to a low R^2.

Num.	hNoV VLPs strain	Proton	App. K_D / mM	R^2
1	GI.1 Norwalk	H7	16.4 ± 3.1	0.9906
		H12	15.2 ± 2.5	0.9927
		H18	19.7 ± 2.8	0.9955
		H19	16.6 ± 2.3	0.9952
		**H21**	**12.2 ± 1.8**	**0.9933**
2	GII.4 Saga	H7	3.8 ± 0.7	0.9689
		H12	2.9 ± 0.8	0.9220
		H18	5.2 ± 0.5	0.9908
		**H19**	**4.7 ± 0.4**	**0.9912**
		H21	5.1 ± 0.6	0.9858
3	GII.4 Ast6139	H12	33.6 ± 10.8	0.9851
		H18	39.7 ± 10.7	0.9914
		H19	33.0 ± 10.1	0.9862
		**H21**	**26.5 ± 8.0**	**0.9830**
4	GII.7 RKI	H7	19.1 ± 6.3	0.9891
		**H12**	**10.9 ± 4.3**	**0.9775**
		H18	27.4 ± 9.8	0.9913
		H19	17.3 ± 5.1	0.9904
		H21	20.6 ± 6.8	0.9900
5	GII.10 Vietnam026	**H7**	**12.9 ± 2.9**	**0.9881**
		H12	15.0 ± 3.0	0.9919
		H18	24.3 ± 6.5	0.9913
		H19	23.5 ± 6.9	0.9891
		H21	17.6 ± 4.2	0.9903
6	GII.17 Kawasaki308	H7	54.1 ± 10.7	0.9975
		H12	69.7 ± 14.2	0.9982
		H18	52.1 ± 6.5	0.9990
		**H19**	**31.5** ± 2.8	**0.9990**
		H21	54.9 ± 8.1	0.9987
7	GII.17 Saitama/T87	H7	8.6 ± 0.3	0.9996
		H12	8.4 ± 0.9	0.9970
		H18	8.5 ± 0.5	0.9989
		**H19**	**6.6 ± 0.4**	**0.9990**
		H21	9.7 ± 1.6	0.9926

Fig. S6 and Table S2 - STD NMR titrations of GII.4 Saga VLPs with CA using initial STD growth rates

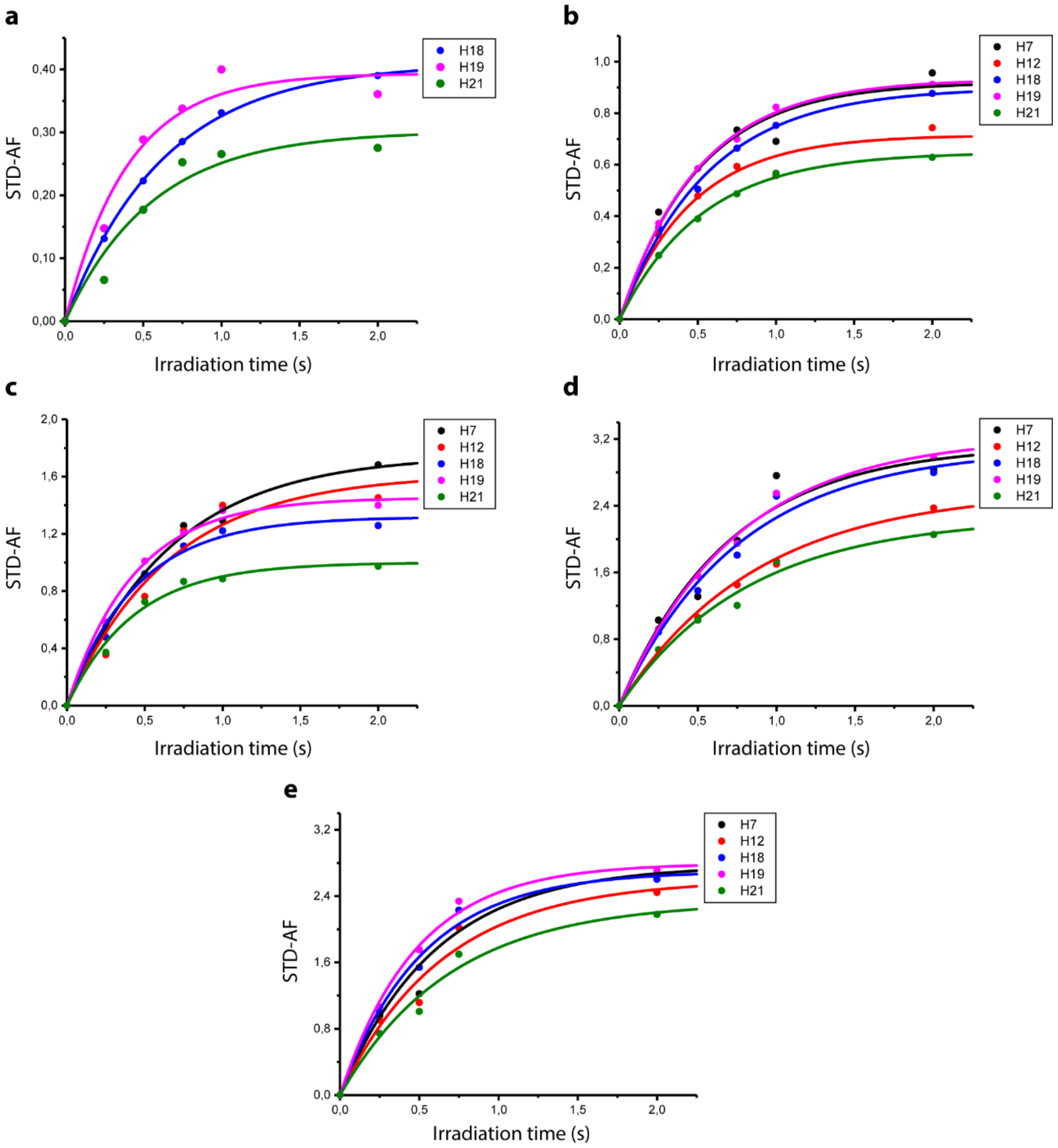

Figure S6: Buildup curves obtained for GII.4 Saga VLPs at concentrations of **a)** 0.5 mM, **b)** 1.5 mM, **c)** 3 mM, **d)** 6 mM and **e)** 9 mM of CA. Shown are STD-AF values as a function of increasing irradiation times. Only isolated signals from CA were selected for analysis. STD NMR experiments were performed at 600 MHz and 277 K.

Table S2: Apparent equilibrium dissociation constants K_D obtained from STD initial growth rates for titration of GII.4 Saga VLPs with CA. The lowest value is highlighted in bold. Compare entry 6 in Table 1 of the main text.

huNoV VLP strain	Proton	App. K_D /mM	R^2
GII.4 Saga 2006	H7	4.5 ± 1.4	0.9845
	H12	4.0 ± 1.3	0.9827
	H18	6.2 ± 1.0	0.9961
	H19	5.4 ± 1.3	0.9891
	**H21**	**3.6 ± 0.7**	**0.9907**

Fig. S7 - Tentative assignments in $^{1}H,^{15}N$ TROSY HSQC spectra of [*U*-$^{2}H,^{15}N$]-labeled MI001 P-dimers

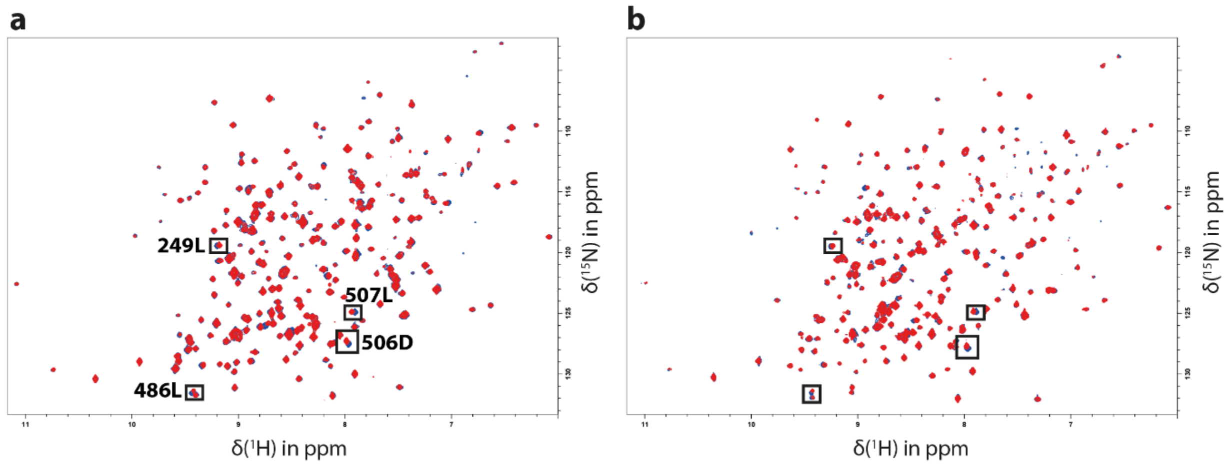

Figure S7: Section of $^{1}H,^{15}N$ TROSY HSQC spectra of **a)** 100 µM [*U*-$^{2}H,^{15}N$] Saga P-dimers and **b)** 100µM [*U*-$^{2}H,^{15}N$] MI001 P-dimers showing the apo protein (blue) and after the addition of 8 mM CA (red). Highlighted are signals showing CSPs and where an assignment can be transferred from Saga to MI001. Spectra were recorded at 500 MHz at 298 K.

Fig. S8 - CSP titration of MIL^{ProS}V^{ProS}A ^{13}C-methyl labeled Kawasaki308 P-dimers with CA (methyl TROSY spectra)

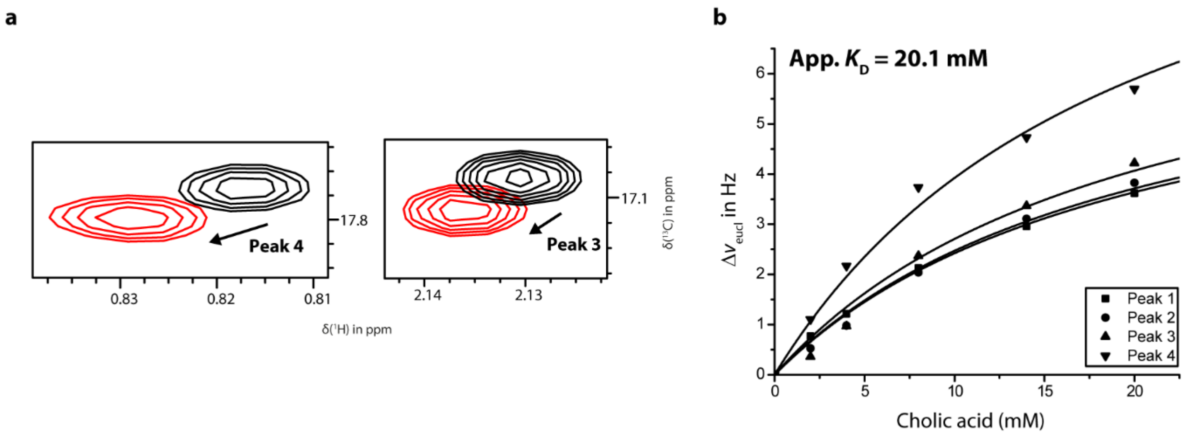

Figure S8: Titration of MIL^{ProS}V^{ProS}A ^{13}C-methyl labeled P-dimers of a GII.17 Kawasaki308 strain with CA. **a)** Representative signals showing CSPs larger than *mean + 2σ* observed in the methyl TROSY experiments at 20 mM cholic acid concentration. **b)** Global fitting of binding isotherms to the CSP data using the law of mass action (cf. Eq. 1 of the main text). The curves reflect one-site binding, and global fitting yields an apparent equilibrium dissociation constant K_D of 20.1 ± 2.9 mM. The location of the binding pocket could not be defined due to the lack of an assignment. Experiments were acquired at 500 MHz and 298 K.

Fig. S9 - Binding isotherm from a CSP titration of [*U*-^{2}H,^{15}N] Saga P-dimers with GCDCA

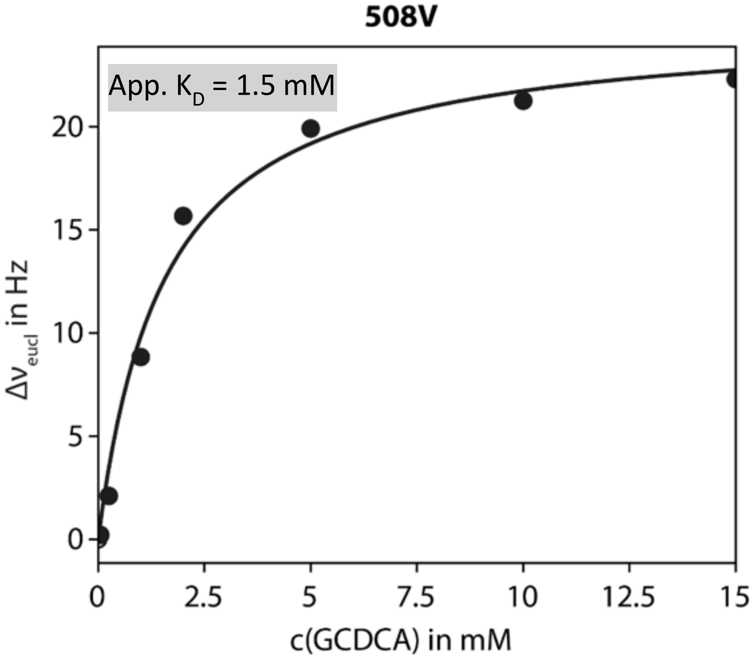

Figure S9: Titration of 50 μM [*U*-^{2}H,^{15}N] Saga P-dimers with GCDCA up to 15 mM in 75 mM sodium phosphate buffer, 100 mM NaCl, pH* 7.3. Fitting of NH signal intensities of V508 using Eq. 1 of the main text furnished an apparent K_D value of 1.5 mM ± 0.25 mM. Experiments were performed at 500 MHz and 298 K.

Fig. S10 - STD NMR spectra of CA in the presence of N373D GII.4 Saga P-dimers

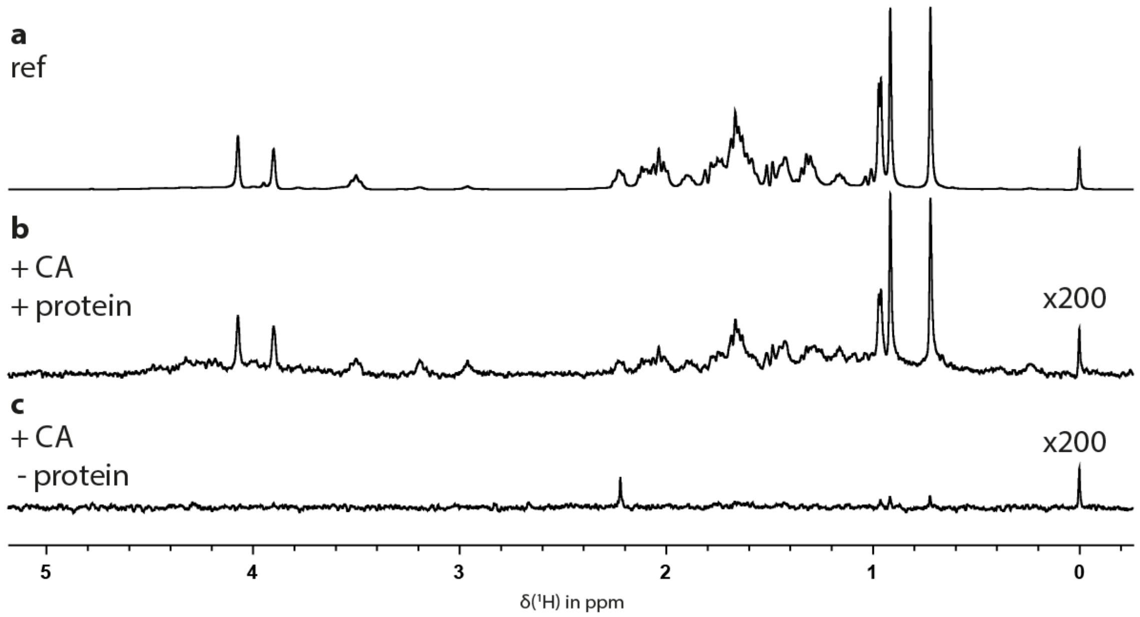

Figure S10: STD NMR spectra showing the interaction of GII.4 Saga N373D P-dimers with CA. **a)** Reference ^{1}H NMR spectrum of CA. **b)** STD NMR spectrum in the presence of 1 mM CA (45 µM of GII.4 373D P-dimers). **c)** Control STD NMR spectrum of CA in the absence of protein. Experiments were performed at 600 MHz and 298 K. N.b.: The STD NMR titration (cf. Table 1 of the main text) was performed at 277 K.

Fig. S11 - Binding of glycyrrhizin (GR) to GII.4 Saga P-dimers and VLPs: CSPs in a $^{1}H,^{15}N$ TROSY HSQC spectrum of [U-$^{2}H,^{15}N$] labeled P-dimers and STD NMR spectrum of CA in the presence of VLPs

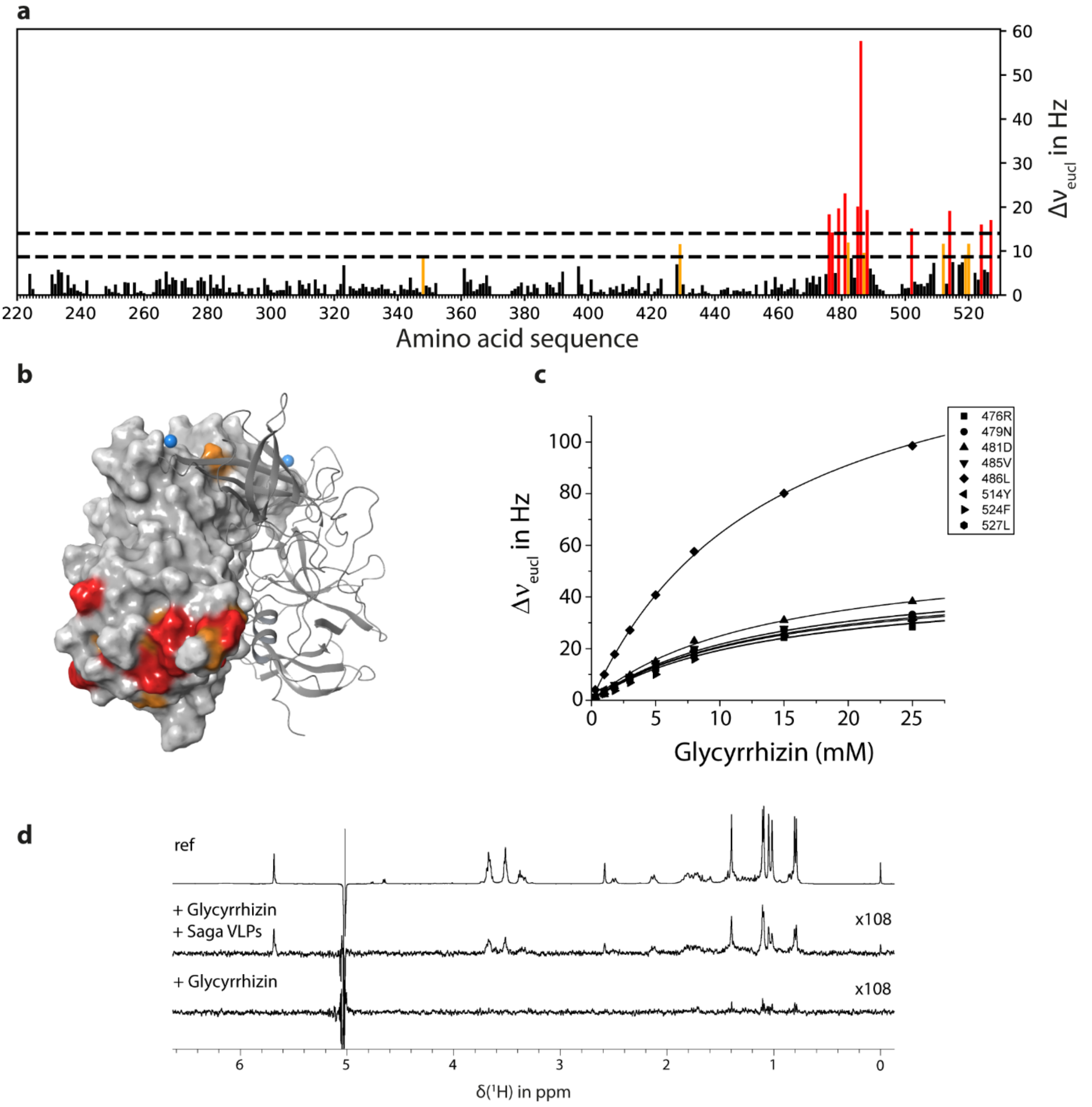

Figure S11: a) Chemical shift perturbations (CSPs as Euclidean distances) of backbone NH signals as a function of amino acid position observed for a [U-$^{2}H,^{15}N$] labeled sample of GII.4 Saga P-dimers (100 μM) in the presence of 8 mM glycyrrhizin (GR). CSPs larger than *mean+σ* are shown in orange and values larger than *mean+2σ* in red. Spectra were acquired at 500 MHz and 298 K. **b)** Mapping of CSPs onto the crystal structure of P-dimers (pdb 4X06) using the same color coding as in (a). The HBGA binding site is highlighted with a blue ball (position of C6 of the fucose moiety of B-trisaccharide). **c)** Binding isotherms from $^{1}H,^{15}N$ TROSY HSQC spectra. Non-linear least squares fitting to Eq. 1 (cf. main text) furnished an apparent K_D value of 13.8 ± 0.4 mM. Only signals showing CSPs larger than *mean+2σ* at 25 mM glycyrrhizin concentration were selected for analysis. **d)** STD NMR experiments showing the interaction of GII.4 Saga VLPs with glycyrrhizin. The panel shows the reference ^{1}H NMR spectrum of glycyrrhizin (*top*), STD NMR spectrum of 2 mM glycyrrhizin in the presence of 0.57 mg/ml VP1 GII.4 Saga VLPs (*middle*) and a control STD NMR spectrum of 2 mM glycyrrhizin (*bottom*). STD NMR spectra were acquired at 600 MHz and 277 K.

Fig. S12 - Backbone dynamics of GII.4 Saga P-dimers from MD

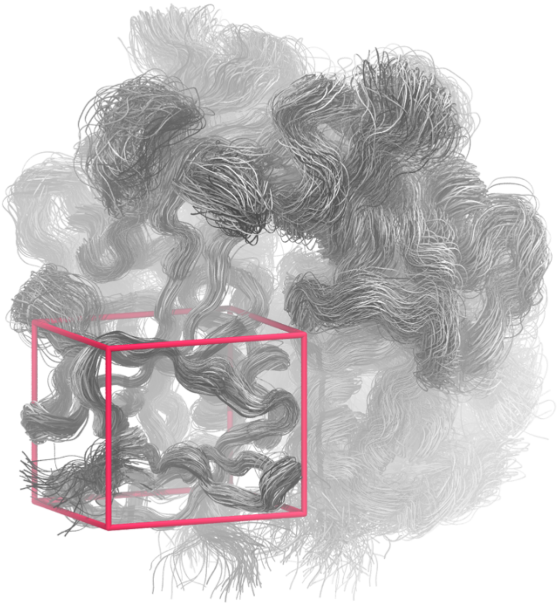

Figure S12: Overlay of ribbon diagrams of 200 out of the 2000 protein conformations of GII.4 Saga P-dimers used for docking. The red box shows the search space. The protein is aligned to the backbone of the amino acids showing significant CSPs.

Fig. S13 - Computation of volume and shape of the binding site

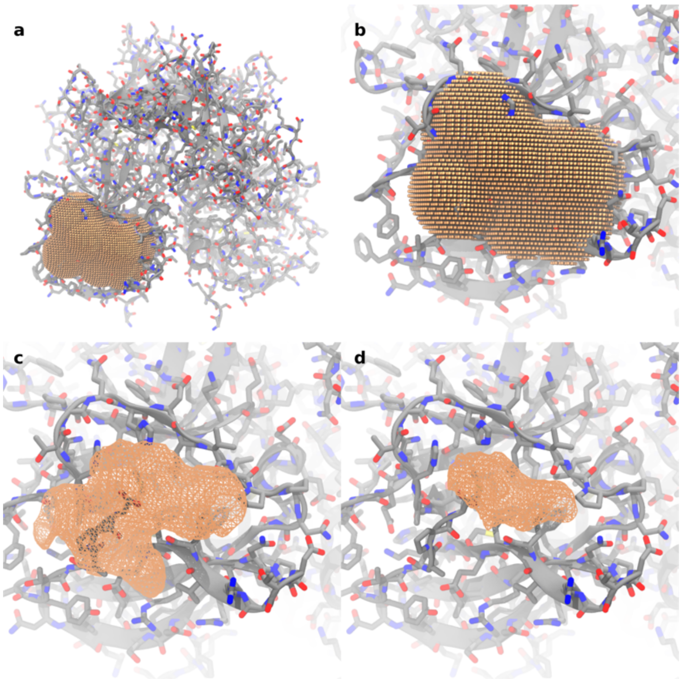

Figure S13: **a)** Image of the whole dimer and the pocket search space located (observed grid points are shown as orange spheres) close to the C-terminus of one of the chains. **b)** Close-up view of the search space. **c)** Accessible volume in at least 10% of the trajectory. **d)** Accessible volume in at least 50 % of the trajectory.

Fig S14 - Docking scores of DCA, CDCA, and GCDCA to an ensemble of GII.4 Saga P-dimers conformations

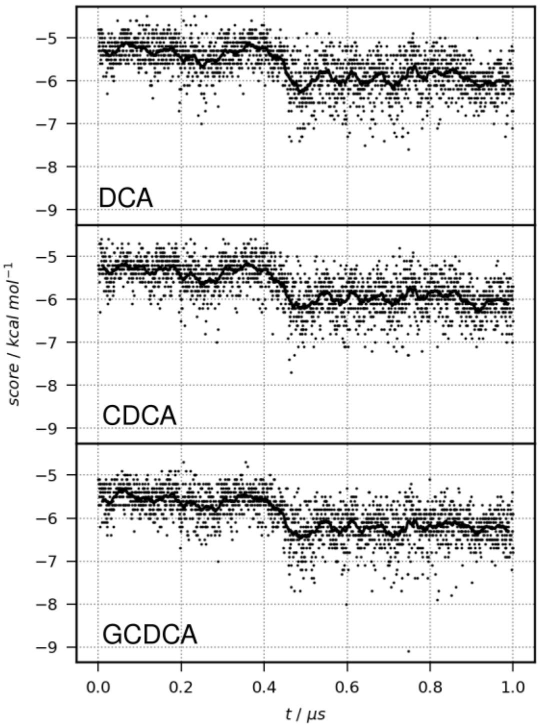

Figure S14: Docking scores of DCA, CDCA, and GCDCA to 2.000 snapshots of the 1 µs MD trajectory of GII.4 Saga P-dimers. The solid line represents a moving average of each 40 points.

Fig. S15 and Table S3- Top five scoring protein-ligand docking poses for CA, DCA, CDCA and GCDCA and docking scores

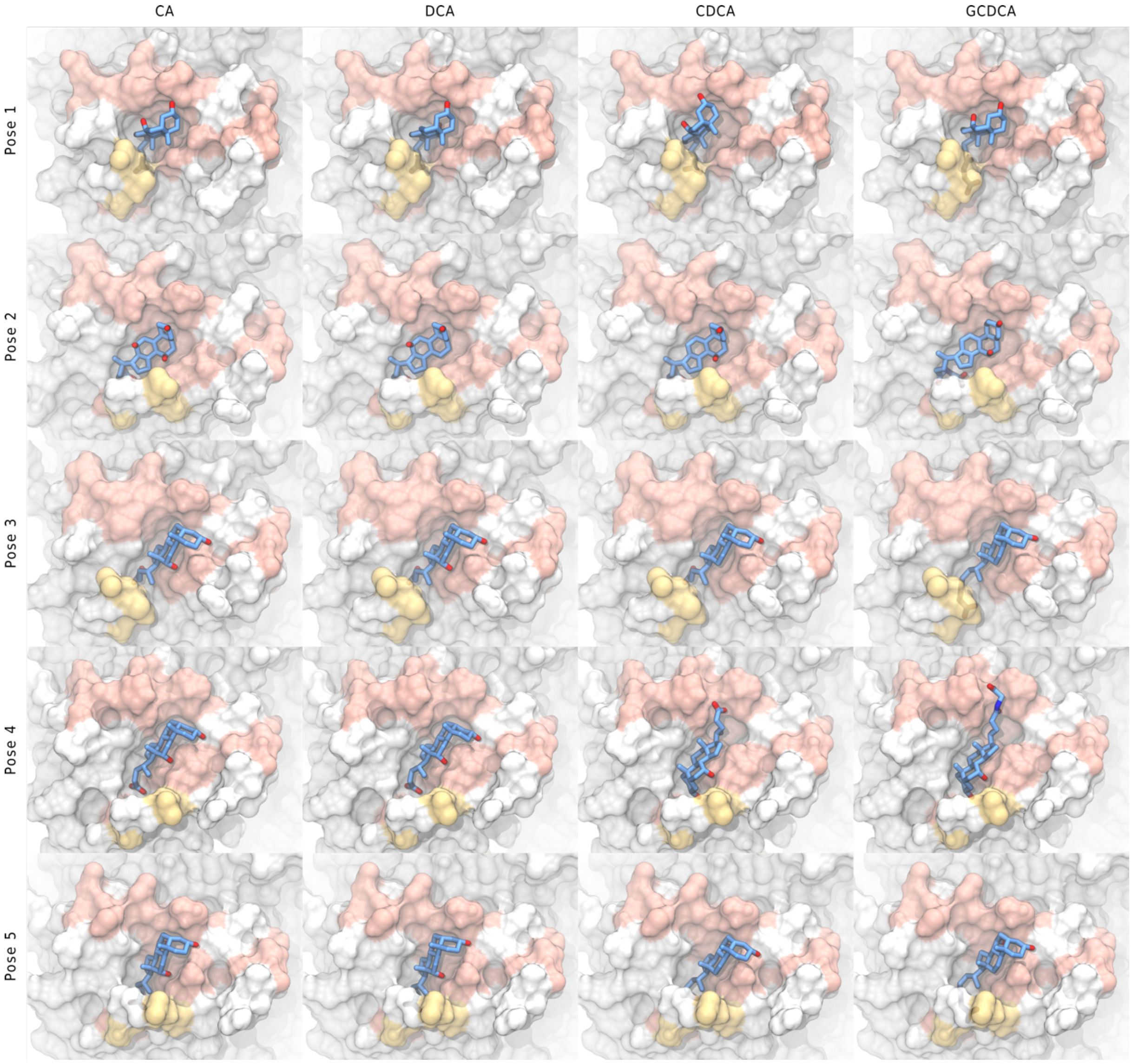

Figure S15: Snapshots of the top five scoring protein-ligand docking poses for each of the 4 bile acids (CA, DCA, CDCA, GCDCA). Protein is shown as solvent-accessible surface. Ligands are drawn in blue licorice representations with the hydrogens omitted for clarity. Colored surface patches denote amino acids with significant CSPs (pink for $^{1}H^{15}N$ and yellow for $^{1}H^{13}C$ CSPs). The surface is slightly translucent so the ligand behind the C-terminus becomes visible.

Table S3: Docking scores in kcal/mol

	Pose 1	Pose 2	Pose 3	Pose 4	Pose 5
CA	-7.6	-7.5	-7.1	-6.9	-7
DCA	-7.6	-7.4	-7.1	-7.3	-7.3
CDCA	-7.3	-7.7	-7.2	-7.4	-7.2
GCDCA	-9.1	-7.6	-7.9	-7.3	-7.3
Average	-7.9	-7.6	-7.3	-7.2	-7.2

Fig. S16 - Ligand RMSD curves over simulation time of the 50 CA:P-dimers complex MD simulations

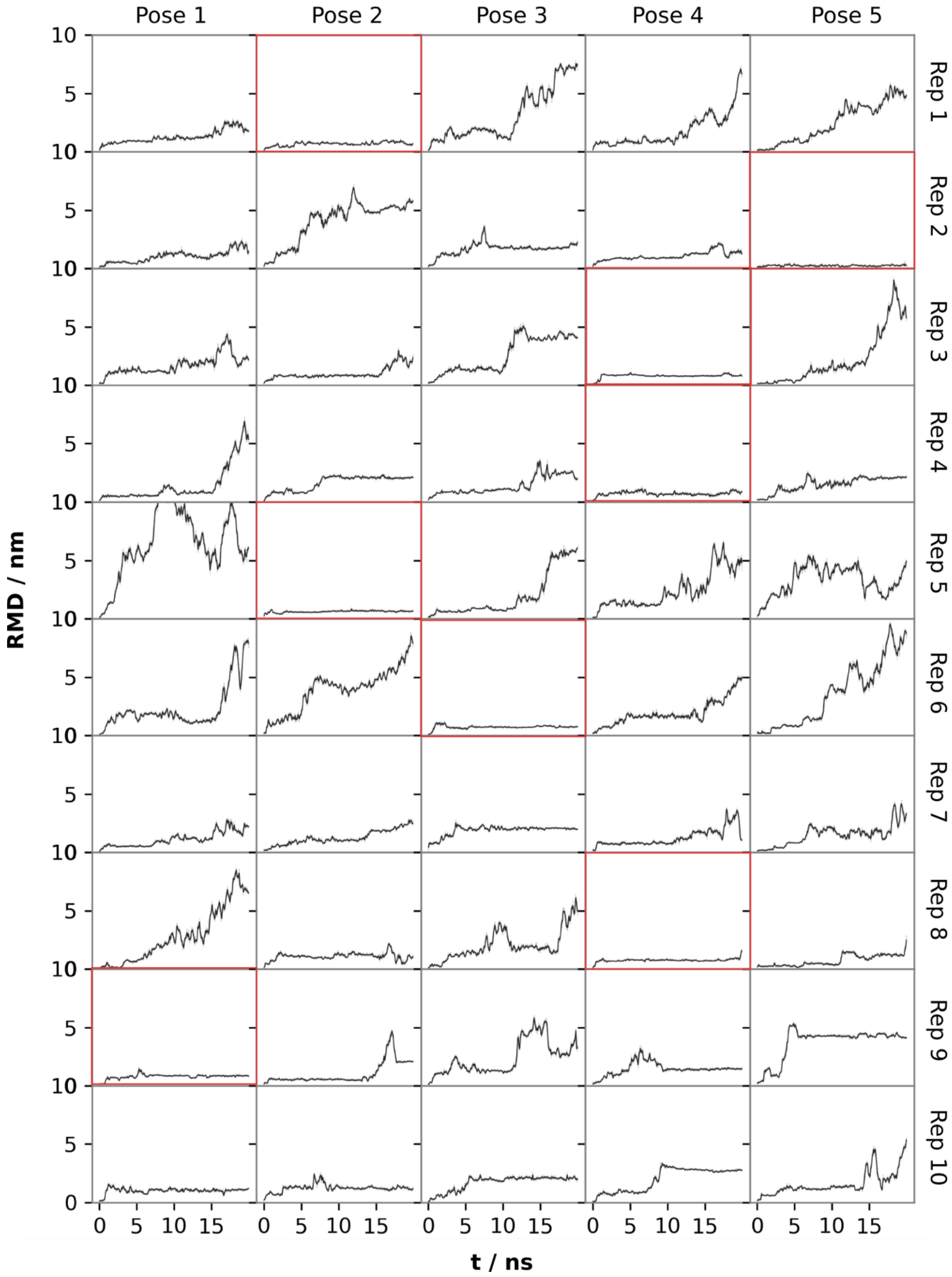

Figure S16: CA ligand RMSDs in nm over simulation time in ns of all the ten replicate simulations for the five initial geometries (docked complexes). The eight trajectories with lowest averages RMSD (last 10 ns) are framed with a red border. The RMSD of only the ligand is considered, with the trajectory being previously fitted to the protein backbone atoms.

Fig. S17 - Contact occupancies between CA and backbone nitrogen atoms

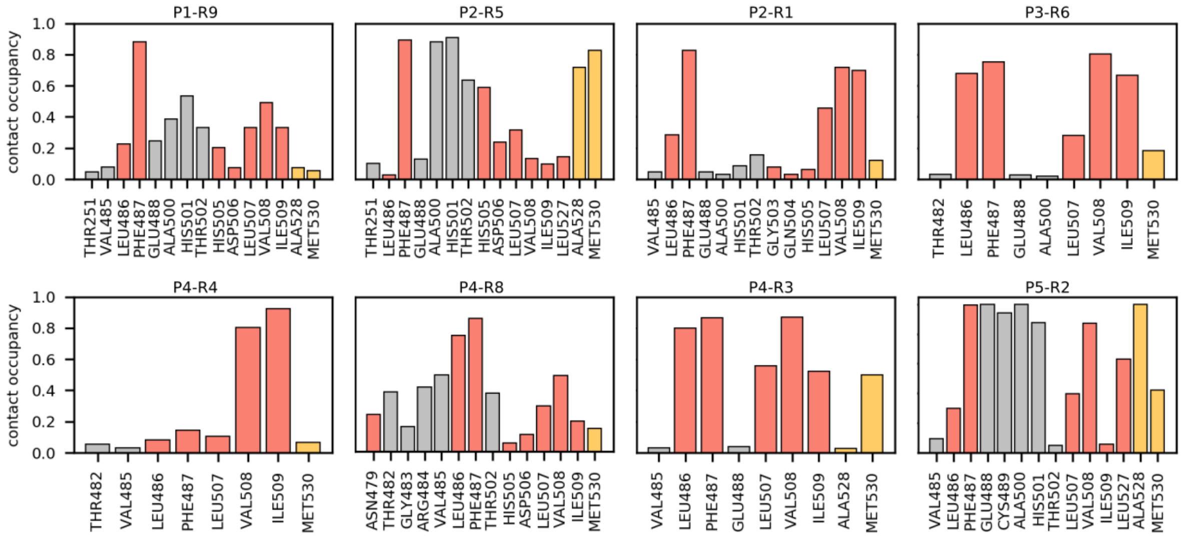

Figure S17: Contact occupancies between CA and backbone nitrogen atoms for the eight simulations with the lowest averaged RMSDs. Contact criterion is a distance ≤ 0.6 nm between the backbone N and at least one heavy atom of CA. Contact amino acids that exhibit significant CSPs are highlighted in red (backbone HSQC) and gold (methyl TROSY), respectively. Proline residues are left out in the analysis. Only amino acids with an occupancy > 0.02 are shown.

Table S4 - Protein biosynthesis of ^{13}C-methyl labeled P-dimers

The solution containing the isotopically labeled precursors (20 ml for 250 ml final medium volume) was prepared as follows: mix 260 mg $Na_2HPO_4*2H_2O$, 72 mg anhydrous KH_2PO_4, 20 mg NaCl, 60 mg deuterated D-glucose (1,2,3,4,5,6,6-d_7), 60 mg NH_4Cl, 9.3 mg of $MgSO_4$, 4 mg of $MgCl_2$, 0.28 mg of $CaCl_2$ and 0.4 mg of vitamin B1 in 5 ml D_2O and lyophilize. Mix the lyophilized powders, the desired labeled amino acids (see Table S4) and 20 µl of the 100x vitamins solution and add D_2O up to 20 ml. Adjust pH* to 7.50, add 100 µg/ml ampicillin and filter under sterile conditions. The medium should be used within the following 24 h after preparation.

Table S4: Precursors used for selective ^{13}C-methyl (MIL^{proS}V^{proS}A) labeling.

Labeled amino acid	Precursor	Quantity per 100 ml culture
Ile	2-keto-3,3-D_2-4-^{13}C-butyric acid[1]	6 mg
Leu^{proS}, Val^{proS}	2-^{13}C-acetolactate[2]	20 mg
Met	L-Methionine-(6-^{13}C)[3]	20 mg
Ala	L-alanine-(3-^{13}C, 2-D)[6]	100 mg
	Succinate-D_4[5]	625 mg

[1]Prepared from 2-keto-4-^{13}C-butyric acid (CortecNet) as explained below. Precursors were obtained from [2]NMR-Bio, [4]Eurisotop, [4,5]Sigma-Aldrich.

Preparation of 2-keto-3,3-D_2-4-^{13}C-butyric acid: 60 mg of 2-keto-4-^{13}C-butyric acid were dissolved in 50 mL D_2O containing 142 mg anhydrous KH_2PO_4 and 240 mg of anhydrous Na_2HPO_4. The pH* was adjusted to 10.5 with NaOD, and the solution was stirred overnight at 45 °C. The H/D exchange was verified by NMR spectroscopy, and the pH* was adjusted to 7.4 with DCl. The solution was sterile filtered into 5 mL aliquots (6 mg 2-keto-3,3-D_2-4-^{13}C-butyric acid each) and stored at -20 °C.

Table S5 - Summary of bile acid binding results with different NoV strains

Table S5: human NoV strains and bile acids for which binding has been detected.

No.	huNoV strain	Protein	Isotopic labeling	Ligand	NMR experiment
10	GI.1 Norwalk	VLPs	-	CA, GCA, TCA, GCDCA, TCDCA	STD
2	GII.4 Saga	P dimers	MIL^{ProS}V^{ProS}A	CA	HSQC-TROSY, Me-TROSY
		P dimers	*u*-[^{2}H,^{15}N]	CA, DCA, GCDCA, CDCA, GR	HSQC-TROSY
		VLPs	-	CA, GCA, TCA, GCDCA, TCDCA, GR	STD
3	GII.4 Saga N373D	P dimers	-	CA	STD
4	GII.4 MI001	P dimers	*u*-[^{2}H,^{15}N]	CA	HSQC-TROSY
5	GII.4 Ast6139	VLPs	-	CA, GCA, TCA, GCDCA, TCDCA	STD
6	GII.7 RKI	VLPs	-	CA, GCA, TCA, GCDCA, TCDCA	STD
7	GII.10 Vietnam026	VLPs	-	CA, GCA, TCA, GCDCA, TCDCA	STD
8	GII.17 Kawasaki308	P dimers	MIL^{ProS}V^{ProS}A	CA	Me-TROSY
		VLPs	-	CA, GCA, TCA, GCDCA, TCDCA	STD
9	GII.17 Saitama/T87	VLPs	-	CA, GCA, TCA, GCDCA, TCDCA	STD

Fig. S18 - Structural alignment of NoV sequences

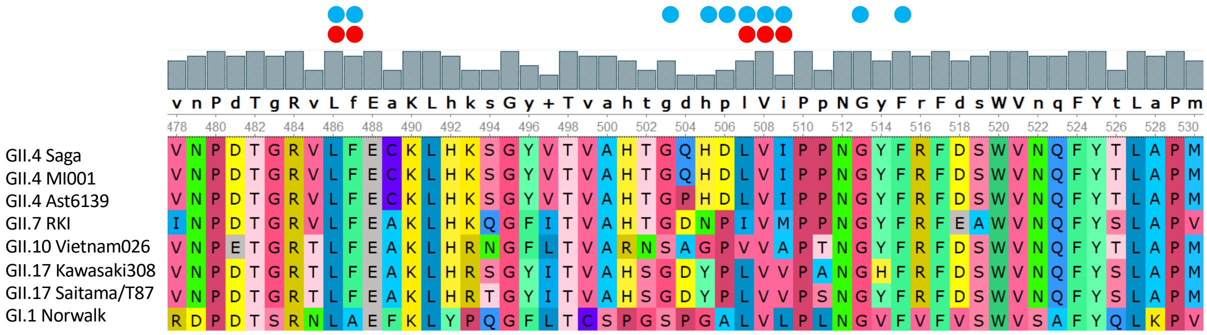

Figure S18: Amino acid sequences of the Norovirus strains studied were subjected to an online structural alignment using the PROMALS3D server (Pei J, Kim BH, Grishin NV: *PROMALS3D: a tool for multiple protein sequence and structure.* Nucleic Acids Res. 36, 2295-2300 (2008). DOI: 10.1093/nar/gkn072). Amino acid positions with predicted bile acid contacts are marked in red (cf. Fig. 8 main text). Positions showing backbone CSPs larger than σ are marked in blue (cf. Fig. 1 main text). It is seen that some of the amino acids directly identified by CSPs in GII.4 Saga to be involved in binding (blue circles), e.g. I509, can vary significantly without impeding binding. The Saga GII.4 strain is used as a reference for the numbering.
